# Multi-omics decodes a defect-interface dual-engineered PtPb@SbO_3-x_ nanozyme for NIR-II photothermal-amplified eradication of drug-resistant pneumonia

**DOI:** 10.1016/j.mtbio.2026.103535

**Published:** 2026-08-05

**Authors:** Zhangwei Qiu, Danyan Wang, Zijun Jin, Jiaping Han, Xueli Wang, Xiaojun He, Liqin Wu

**Affiliations:** a Department of Respiratory and Critical Care Medicine, The Second Affiliated Hospital and Yuying Children's Hospital of Wenzhou Medical University, Wenzhou, Zhejiang, 325000, China; b Zhejiang Chinese Medical University, Hangzhou, Zhejiang, 310000, China; c Department of Anesthesiology, The First Affiliated Hospital of Wenzhou Medical University, Wenzhou, Zhejiang, 325000, China; d Department of Ophthalmology, Peking University First Hospital, Beijing, 100034, China; e Zhejiang Key Laboratory of Smart Biomaterials and Center for Bionanoengineering, College of Chemical and Biological Engineering, Zhejiang University, Hangzhou, 310058, China

**Keywords:** Defect engineering, Nanozyme, Multi-omics, Drug-resistant bacterial, Pneumonia

## Abstract

Managing pneumonia caused by multidrug-resistant (MDR) bacteria presents significant clinical challenges. The near-infrared II (NIR-II) laser irradiance exhibits strong photothermal conversion capabilities, making it a promising candidate for photothermal and chemodynamic therapies as non-antibiotic strategies. However, traditional methods are often hindered by issues such as the uncontrollable production of reactive oxygen species and the low efficiency associated with NIR-II photothermal therapy. This underscores the necessity for precisely regulated and highly effective synergistic therapies. To address these limitations, we have innovatively developed a defect/interface dual-engineered nanozyme (PtPb@SbO_3-x_) and comprehensively characterized its atomic-scale structure and catalytic mechanisms using density functional theory calculations and synchrotron radiation techniques. *In vitro* experiments demonstrated that PtPb@SbO_3-x_ could efficiently eliminate drug-resistant bacteria and disrupt biofilm structures under low-concentration hydrogen peroxide and NIR-II irradiation while exhibiting excellent biocompatibility. In pneumonia models, the nanozyme enabled rapid infection clearance and significantly reduced inflammatory responses via synergistic photothermal and chemodynamic therapy effects. Furthermore, integrated multi-omics analyses-including metabolomics, transcriptomics, and proteomics-systematically uncovered the molecular mechanisms driving its therapeutic efficacy. This study successfully establishes a non-antibiotic nanozyme-based therapeutic strategy that is efficient, low in toxicity, and non-invasive for treating MDR bacterial infections. It also provides a solid theoretical basis and technical framework for the rational design of defect/interface dual-engineered nanoplatforms.

## Graphical abstract

**Figure:**
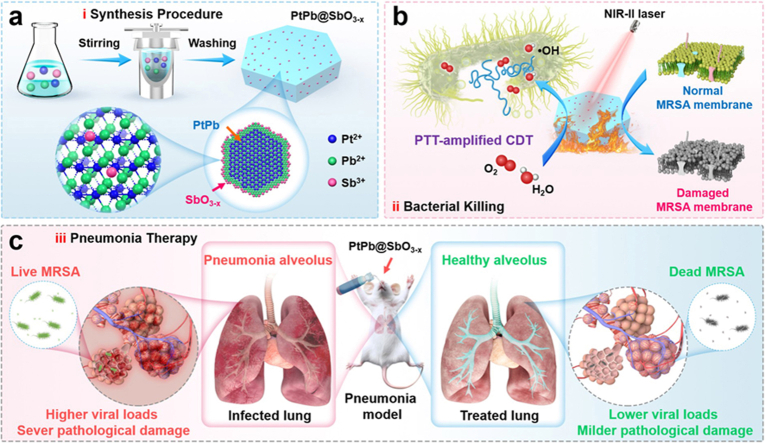

## 1 Introduction

**Scheme 1:**
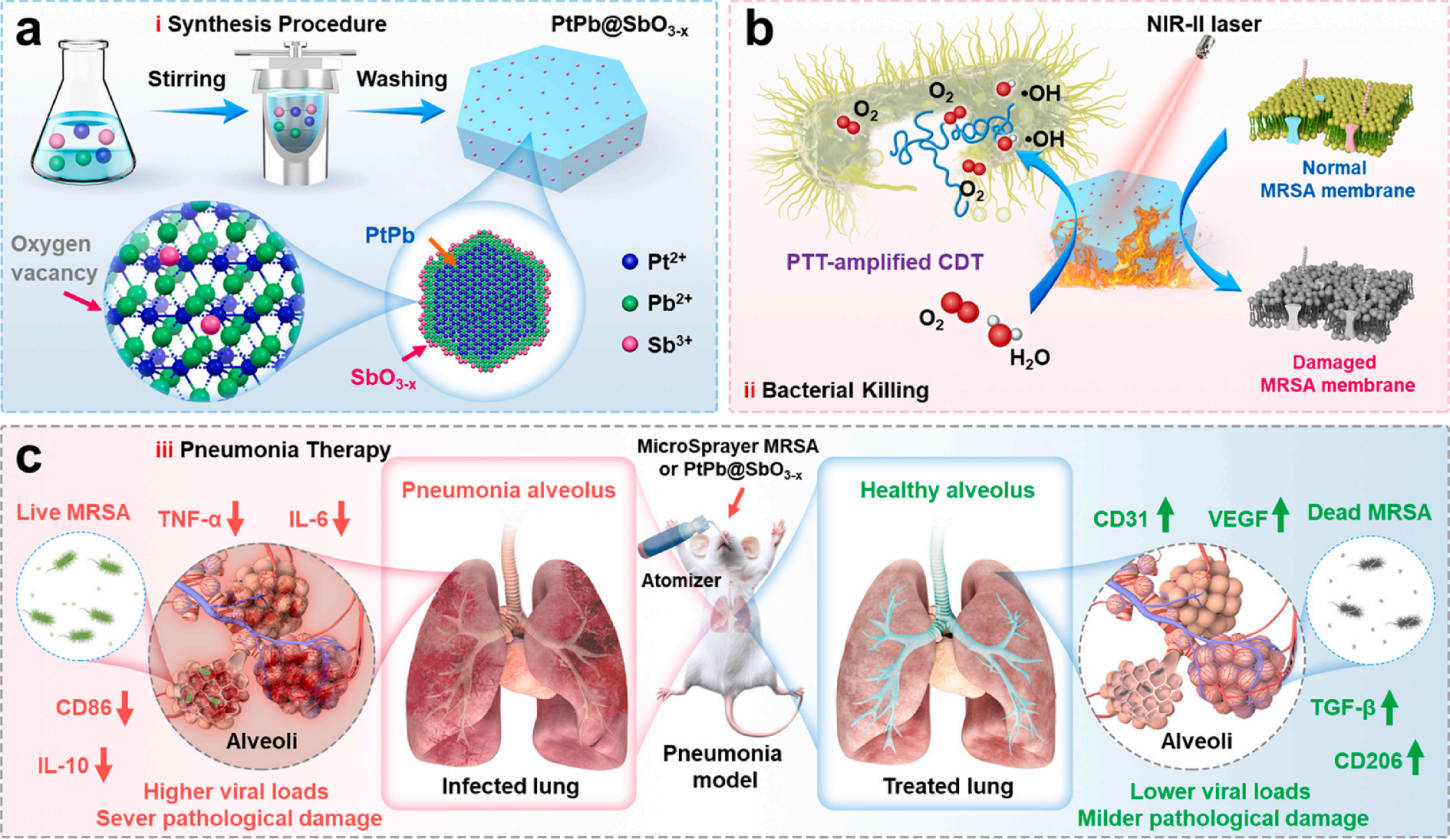
PtPb@SbO_3-x_ nanoplatforms for MRSA-infected pneumonia therapy. (a) The fabrication process of PtPb@SbO_3-x_ nanozymes through hydrothermal synthesis and surface functionalization. (b) Multimodal anti-biofilm mechanism: (i) Peroxidase-mimetic activity generates ROS, (ii) ROS-triggered biofilm matrix degradation, and (iii) enhanced nanozyme penetration through biofilm permeability barrier disruption. (c) Therapeutic workflow for MRSA-infected pneumonia: (i) Synergistic antibiofilm/antibacterial action through ROS generation and hyperthermia and (ii) Concomitant anti-inflammatory effects and macrophage reprogramming for lung tissue healing.

Multidrug-resistant (MDR) bacteria, which are primary pathogens responsible for hospital-acquired infections, have emerged as a significant global public health crisis [1]. These pathogens not only cause life-threatening complications, such as invasive pneumonia and septicemia, particularly in immunocompromised patients, but they also perpetuate a vicious cycle of escalating drug resistance and infection rates due to their dynamic mechanisms of resistance acquisition and characteristics of nosocomial transmission. According to the latest statistics from the World Health Organization (WHO), antimicrobial resistance is directly responsible for approximately 1.27 million deaths annually, with MDR bacterial pneumonia accounting for 38% of these cases, thereby placing substantial strain on healthcare systems [2]. Despite increased investments in new antibiotic development, known mechanisms of bacterial resistance, including horizontal gene transfer and biofilm formation, are evolving at a supralinear rate, outpacing drug development [3]. Therefore, there is an urgent need to shift towards non-antibiotic antimicrobial approaches that can circumvent resistance mechanisms (see Scheme 1).

The rise of nanomedicine has ushered in revolutionary techniques to overcome resistance. Nanomaterials that exhibit functions similar to enzymes and modulate the metabolism of reactive oxygen species (ROS) are gradually altering anti-infective strategies [[4], [5], [6]]. Strategies based on chemodynamic therapy (CDT), utilizing Fenton/Fenton-mimetic chemistry offer several advantages. A unique benefit of such strategies is that the cytotoxic hydroxyl radical (•OH) is generated by converting endogenous hydrogen peroxide (H_2_O_2_) at infected sites through pathogen targeting. Furthermore, nanozyme activity can overcome the inherent limitations of tissue penetration associated with conventional phototherapies [7]. Additionally, multi-enzyme by-products can simultaneously disrupt of bacterial membranes, degrade biofilms and damage DNA [8]. To date, more than 50 classes of nanozyme systems have been developed, including noble metal alloys and metal-organic frameworks that exhibit catalytic efficiencies 2-3 orders of magnitude higher than natural enzymes. Nanozymes based on platinum possess a distinct d-band electronic structure, enabling them to exhibit 4 catalytic activities: oxidase (OXD) [9], peroxidase (POD) [10], catalase (CAT), and superoxide dismutase (SOD) [11]. This characteristic allows them to function as a “molecular switch”, toggling between pro-oxidative (OXD/POD) and antioxidative (CAT/SOD) modes, thereby facilitating exquisite regulation of ROS [12,13].

This paper explores the microenvironment of deep tissue infections by utilizing a novel strategy based on electronic structure engineering and interface defect modulation to create a multifunctional nanozyme system (PtPb@SbO_3-x_). The core of the PtPb solid solution, produced through atomic layer deposition, leverages the p-orbital electron filling of Pb to optimize the d-band center position of Pt, significantly enhancing the adsorption energy of H_2_O_2_. An SbO_3-x_ shell with oxygen vacancies is introduced at the interface, leading to the formation of a Schottky heterojunction that facilitates the segregation of photogenerated carriers and the formation of localized electric fields, thereby promoting the polarization and cleavage of H_2_O_2_ [14]. The system demonstrates strong photothermal conversion efficiency and POD-mimetic activity under 1064 nm laser irradiation, resulting in the continuous release of •OH in the presence of H_2_O_2_. The study indicates that PtPb@SbO_3-x_ possesses dual functions for bacterial eradication, as evidenced in murine models infected with methicillin-resistant *Staphylococcus aureus* (MRSA). The catalytic material effectively eliminates biofilms and other bacteria through cascade catalysis. Furthermore, the photothermal therapy (PTT) effect kills the bacteria and enhances membrane permeability the membrane. This boosts the work of ROS [15,16]. The bacterial load in the lungs of treated mice exhibited a 3-log decrease. Additionally, these treated mice demonstrated significant histopathological recovery without systemic toxicity. This study addresses the limitations of single-modality nanozyme therapies against MDR infections and establishes a multifunctional platform that was rationally designed to combat MDR infections in deep tissues. The excellent catalytic performance and biosafety of the PtPb@SbO_3-x_ system will provide not only theoretical support for the design of anti-infective nanozymes but also a solid foundation for clinical translation. The mechanistic insights gained will pave the way for the rational design of intelligent nanozyme platforms with microenvironment-responsive specificity, adaptive catalytic switching capabilities, and precise spatiotemporal control, which will undoubtedly revolutionize targeted antimicrobials through enzyme-mimetic precision therapeutics.

## 2 Experimental section

*Materials and Instruments.* Platinum (II) acetylacetonate (Pt(acac)_2_), Lead (II) acetylacetonate (Pb(acac)_2_), Antimony (III) acetate (Sb(Ac)_3_), L-ascorbic acid (AA), salicylic acid (SA), oleylamine (OAm), 1-octadecene (ODE), 3,3′,5,5′-tetramethylbenzidine (TMB), o-phenylenediamine (OPD), and sodium acetate were procured from Aladdin (Shanghai, China). Tryptic soy broth (TSB), LB broth, and agar powder were obtained from Solarbio (Beijing, China). The LIVE/DEAD® BacLight™ Bacterial Viability Kit was obtained from Thermo Fisher Scientific (Waltham, USA). Phosphate-buffered saline (PBS, pH 7.4) and Dulbecco's Modified Eagle Medium (DMEM, high glucose) were acquired from Thermo Scientific (Suzhou, China). A transmission electron microscope (TEM, Talos F200S) operating at 200 kV was employed for the morphological characterization of PtPb@SbO_3-x_. The analysis of elemental composition and valence states was performed using X-ray photoelectron spectroscopy (XPS, Thermo ESCALAB nexsa G2) and Raman spectroscopy (HORIBA HR Evolution). UV-visible-NIR-II absorption spectra were recorded using a Cary 5000 spectrophotometer (Agilent). Confocal laser scanning microscopy (CLSM, Nikon A1, Japan) was utilized for fluorescence imaging. XAFS data were collected at the BL20U station of the Shanghai Synchrotron Radiation Facility (SSRF), operating at an energy of 3.5 GeV with a current of 210 mA. The XAFS spectral data were obtained at the Pt L_3_-edge and calibrated using Pt foil and PtO_2_ standard samples.

*Synthesis of PtPb@SbO*_*3-x*_. The synthesis procedure of PtPb@SbO_3-x_ nanozyme is delineated as follows: Initially, Pt(acac)_2_ (5.0 mg), Pb(acac)_2_ (4.0 mg), Sb(Ac)_3_ (3.0 mg), and AA (25.0 mg) were accurately weighed and introduced into a 50 mL three-neck flask, along with the surfactant OAm (5.0 mL) and ODE (2.0 mL). The mixture underwent ultrasonic dispersion treatment (100 W, 40 kHz) for 1 h using an ultrasonic cleaner until a homogeneous reddish-brown solution was formed. Following the ultrasonic dispersion, the three-neck flask was continuously purged with high-purity argon at a flow rate of 20 mL/min to eliminate air. During the heating, holding, and natural cooling phases, the argon flow was maintained consistently until the reaction was complete. Subsequently, the reaction system was placed under an argon atmosphere in a preheated oil bath set to 200^°^C for thermal decomposition, with continuous stirring at 600 rpm for a duration of 6 h. Upon completion of the reaction, heating was ceased, and the system was allowed to cool naturally to room temperature in an argon environment. Upon completion of the reaction, heating was ceased, and the system was allowed to cool naturally to room temperature. The products were collected via centrifugation at 8000 rpm for 5 min. To eliminate unreacted precursors and organic residues, the obtained precipitates were washed five times with a cyclohexane/ethanol mixed solution (v/v = 2:8) through repeated centrifugation. Finally, the resulting black solids were stored in a vacuum-drying oven for subsequent use. The average yield of the obtained black solid was 78.3%, based on the total metal precursors (Pt, Pb, and Sb). The synthesis was repeated three times, yielding consistent results [17,18].

*Photothermal Characterization and Stability Profiling of PtPb@SbO*_*3-x*_*.* Real-time thermal imaging was acquired using a FLIR T865 infrared camera, which has a measurement accuracy of ±1%, at a sampling frequency of 25 Hz. For concentration-dependent studies, aqueous dispersions of PtPb@SbO_3-x_ (ranging from 25 to 100 ppm in PBS) were placed in 96-well plates and irradiated with a 1064 nm laser at an intensity of 1 W/cm^2^ for 6 min. The dependency on power density was assessed by exposing a fixed concentration dispersion (100 ppm) to varying intensities (from 0.25 to 1.5 W/cm^2^). Thermal profiles were analyzed using FLIR Research Studio software (version 3.4). The operational durability of the nanocomposite was evaluated through five consecutive heating-cooling cycles, with each cycle consisting of 6 min of NIR-II irradiation at 1 W/cm^2^ followed by 10 min of passive cooling to ambient temperature (25 ± 0.5°C). The photothermal conversion efficiency (*η*) was calculated using a modified version of Roper's method. Upon reaching thermal equilibrium, the laser was deactivated to monitor the cooling kinetics. The time constant (τs) was derived from the exponential fitting of the cooling phase.

τs=−t/ln(θ)

where

$$\theta =\left(T_{\left(t\right)}-T_{\text{surr}}\right)/\left(T_{\max}-T_{\text{surr}}\right)*$$

Conversion efficiency was calculated using:

$$\eta =\left[\text{hS}\left(T_{\max}-T_{\text{surr}}\right)-Q_{\text{dis}}\right]/\left(I\left(1-1_{0}^{-A}\right)\right)$$

Where h = heat transfer coefficient, S = irradiation area, Q_dis_ = PBS solvent heating, I = incident power, and A = absorbance at 1064 nm.

*Antimicrobial Efficacy Assessment.* Methicillin-resistant *Staphylococcus aureus* (MRSA, ATCC 43300) was cultured in Tryptic Soy Broth (TSB) at 37^°^C. Bacterial suspensions were standardized to an optical density (OD600) of 0.1 (approximately 1 × 10^8^ CFU/mL) and subsequently diluted to 1 × 10^6^ CFU/mL in sterile PBS. Eight experimental groups (n = 3/group) were established in 96-well plates: (1) MRSA; (2) MRSA + NIR-II; (3) MRSA + H_2_O_2_; (4) MRSA + NIR-II + H_2_O_2_; (5) MRSA + PtPb@SbO_3-x_; (6) MRSA + PtPb@SbO_3-x_ + H_2_O_2_; (7) MRSA + PtPb@SbO_3-x_ + NIR-II; (8) MRSA + PtPb@SbO_3-x_ + H_2_O_2_ + NIR-II. NIR-II irradiation was performed using a fiber-coupled laser system equipped with real-time temperature monitoring. Following treatment, 20 μL aliquots from serial dilutions were plated on TSB and incubated at 37°C for 18 h. Colony-forming units were enumerated using an automated colony counter. Bacterial viability was calculated using the formula: *Survival* (%) = (*CFU*_*treatment*_*/CFU*_*control*_) × *100*.

*Bacterial Viability Visualization.* Live/dead staining was performed using the LIVE/DEAD® BacLight™ Kit (Thermo Fisher). Bacterial pellets, which had been treated by centrifugation 8000 × g for 5 min at 4°C were resuspended in 1× PBS. They were stained in the dark with SYTO 9 (3.34 μM) and propidium iodide (20 μM) for 15 min. Fluorescence images were captured using a Nikon A1R HD25 confocal microscope, utilizing the SYTO 9 channel (excitation at 488 nm/emission at 500-550 nm) and the PI channel (excitation at 561 nm/emission at 570-620 nm).

*Ultrastructural Analysis* via *SEM.* Bacterial suspensions were immersed in 0.1 M phosphate-buffered saline (PBS, pH 7.4) and subjected to primary fixation using 2.5% (v/v) glutaraldehyde at 4°C for 12 h to ensure adequate cross-linking. Following fixation, the samples were dehydrated through a stepwise ethanol gradient (30%, 50%, 70%, 90%, and 100%), with a 10-min incubation at each concentration to facilitate gradual solvent replacement. Dehydrated samples were processed using a Leica EM CPD300 critical point dryer with liquid CO_2_ as the transition medium. Subsequently, a conductive coating was applied using a Quorum Q150T ES ion sputter coater, which deposited a 10 nm-thick gold layer under a vacuum level exceeding 5 × 10^−2^ mbar. Metalized specimens were then loaded into a Zeiss Sigma 500 field-emission scanning electron microscope (FE-SEM) chamber. Surface morphology was examined at an accelerating voltage of 5 kV and a working distance of 8-10 mm. High-resolution images were acquired with a resolution better than 1.3 nm at 15 kV, systematically documenting ultrastructural alterations in bacterial surfaces under varying experimental conditions.

*Quantitative Assessment of Biofilm Inhibition.* Biofilms of the MRSA strain were established in 24-well polystyrene plates according to standardized protocols. Overnight cultures were adjusted to an optical density (OD600) of 0.05 in fresh TSB supplemented with 1% glucose to enhance biofilm formation. Each well received 20 μL of bacterial inoculum (resulting in a final volume of 2 mL) followed by static incubation at 37°C for 24 h. After incubation, biofilm-embedded wells underwent three gentle washes with sterile PBS (pH 7.4) to remove planktonic cells. The experimental groups included: Negative control: 200 μL PBS; Oxidative control: 0.1 mM H_2_O_2_; Nanomaterial treatment: 100 ppm PtPb@SbO_3-x_ dispersion; CDT + PTT: PtPb@SbO_3-x_ + H_2_O_2_ + 1064 nm laser (1 W/cm^2^, 5 min irradiation). Following 2 h treatment at 37°C, biofilms were fixed with 200 μL methanol (15 min), then stained with 0.5% (w/v) crystal violet (CV) for 20 min. Excess stain was removed by washing three times with PBS. The CV-bound biofilm matrices were solubilized with 200 μL 33% glacial acetic acid (v/v) under orbital shaking (150 rpm, 10 min). Absorbance was measured at 590 nm using a microplate reader, with biofilm inhibition percentage calculated relative to untreated controls.

*Cytokine Profiling in Bronchoalveolar Lavage Fluid (BALF).* BALF was performed following standard protocols. Lungs from euthanized mice in each experimental group were instilled with 1.5 mL of ice-cold sterile phosphate-buffered saline (PBS, pH 7.4) via tracheal cannulation. The lavage fluid was immediately centrifuged at 2400 rpm for 10 min at 4°C) to separate cells from soluble fractions. The supernatant was aliquoted and stored at −80°C for cytokine analysis, while the pellet was suspended in sterile PBS for cell profiling. The study measured inflammatory mediators, including transforming growth factor-beta, interleukin-10, interleukin-6 and tumor necrosis factor-alpha, using commercial ELISA kits (Neobioscience, China) according to the manufacturer instructions. Absorbance measurements were conducted in triplicate on a SpectraMax M5 microplate reader (Molecular Devices), with the appropriate detection settings for each wavelength (refer to the kit for individual cytokine specifications). Cytokine concentrations were normalized to total protein content, which was measured using the BCA assay (Pierce™, Thermo Fisher Scientific).

*Cell Culture and CCK-8 Cytotoxicity Test.* Human bronchial epithelial cells (BEAS-2B) were cultured in DMEM high glucose medium supplemented with 10% fetal bovine serum and 1% penicillin-streptomycin at 37^°^C in a 5% CO_2_ incubator, with routine passaging. Cells in the early log phase were seeded in 96-well plates at a density of 1 × 10^4^ cells per well. After adherence, cells were treated with PtPb@SbO_3-x_ dispersions in PBS at final concentrations of 0, 25, 50, 100, 150, and 200 ppm for 24 h. Some wells were subsequently exposed to a 1064 nm laser (1.0 W/cm^2^, 6 min). Afterward, 10 μL of CCK-8 reagent was added to each well and incubated for 2 h, followed by measurement of absorbance at 450 nm using a microplate reader. Cell viability was calculated relative to an untreated control, which was set at 100%. All experiments were independently repeated three times (n = 3), and the data are presented as mean ± SD.

*Hemocompatibility Evaluation of PtPb@SbO*_*3-x*_. The hemolytic potential was systematically evaluated using erythrocytes isolated from male BALB/c mice (*Mus musculus*). Fresh whole blood samples were subjected to gradient centrifugation (3000 rpm, 10 min, 25°C) for erythrocyte isolation. The cellular pellets were subsequently washed thrice with phosphate-buffered saline (PBS, pH 7.4) and resuspended in PBS to prepare a 5% (v/v) erythrocyte stock solution, which was maintained at 4°C until experimental use. For hemolysis testing, 500 μL aliquots of the erythrocyte suspension were incubated with equal volumes of PtPb@SbO_3-x_ dispersions at graded concentrations (ranging from 5 to 100 ppm) in 1.5 mL Eppendorf tubes. Parallel control groups were established using deionized water (positive control) and PBS (negative control). All experimental sets were incubated under static conditions at 37°C for 6 h in a temperature-controlled dry bath.

Samples were centrifuged (12,000 rpm, 10 min) to pellet intact erythrocytes. The hemoglobin release was quantified by measuring supernatant absorbance at 540 nm using a microplate reader. The percentage of hemolysis was calculated through normalized comparison using the formula provided

$$Hemolysis\;\left(\%\right)=\left[\left(A_{sample}-A_{negative}\right)/\left(A_{positive}-A_{negative}\right)\right]\times 100$$

Where *A*_*sample*_, *A*_*negative*_, and *A*_*positive*_ represent absorbance values of test groups, PBS control, and DI water control, respectively.

*POD-Mimetic Activity Profiling of PtPb@SbO*_*3-x*_*.* The chromogenic oxidation of TMB and OPD allowed for the calculation of the catalytic production of •OH. The spectra of these substrates change upon oxidation, with TMB yielding a blue chromogen (*λ*_max_ = 652 nm) and OPD producing a yellow-orange product (*λ*_max_ = 420 nm). The reaction systems, consisting of PtPb@SbO_3-x_ (10 ppm), TMB (0.1 mM), and H_2_O_2_ (0.1 mM) in PBS (10 mM, pH 5.0), were incubated under static conditions at 25°C. The UV-vis-NIR spectra were recorded every 0-15 min to monitor the progress over time. Parallel studies assessed laser-enhanced catalysis using irradiation (1064 nm, 1.0 W/cm^2^) during incubation. Complementary assays employed OPD (0.1 mM) with PtPb@SbO_3-x_ (10 ppm) and H_2_O_2_ (0.1 mM) in PBS. To exploit photothermal-enhanced catalytic effects, NIR-II-assisted reactions (1.0 W/cm^2^, 5 min) were conducted. Michaelis-Menten parameters were determined at varying concentrations of H_2_O_2_ (1-12 mM) while maintaining fixed concentrations of PtPb@SbO_3-x_ (10 ppm) and TMB (0.1 mM). The initial reaction velocities (V0) were calculated by linear regression of absorbance alterations at 652 nm within the first 2 min. Kinetic constants (K_m_, V_max_) were calculated using the Lineweaver-Burk transformation: 1/V_0_ = (K_m_/V_max_)(1/[S]) + 1/V_max._ [19].

*Antimicrobial Evaluation in MRSA-Induced Pneumonia Model.* A model of MRSA-associated pneumonia was established using 6-8 week-old female BALB/c mice (20-25 g) following IACUC-approved protocols. Following isoflurane anesthesia (3% induction, 1.5% maintenance), 50 μL of MRSA suspension (2 × 10^7^ CFU/mL in sterile PBS) was administered via orotracheal intubation using a 24G angiocatheter. Mice were maintained in a 45° upright position for 2 min post-inoculation to enhance pulmonary deposition. At 4 h post-infection, animals were randomized into four therapeutic cohorts (n = 5/group): (I) Control: PBS + 1064 nm laser; (II) PTT: PtPb@SbO_3-x_ (100 ppm) + 1064 nm laser; (III) CDT: PtPb@SbO_3-x_ (100 ppm) + H_2_O_2_ (0.1 mM); (IV) PTT + CDT: PtPb@SbO_3-x_ + H_2_O_2_ + 1064 nm laser. Therapeutic agents (200 μL) were delivered via aerosolized intratracheal administration using a MicroSprayer® aerosolizer. NIR-II laser irradiation (1064 nm, 1 W/cm^2^, 5 min) was performed with continuous thermal monitoring using an IR camera (FLIR A65). Initially, five mice were enrolled in each group for the *in vivo* experiments. For subsequent histopathological, immunohistochemical, and biochemical assays (including bacterial burden and H&E staining), we randomly selected three mice from the original five per group for analysis, as these endpoint analyses are resource- and time-intensive. Furthermore, three biological replicates are considered sufficient for statistical inference due to the homogeneity of variance and normal distribution. This approach adheres to the 3Rs principle. The randomization and selection procedures were conducted using a computer-generated random sequence. All animal experimentation was carried out in accordance with the Guidelines for Care and Use of Laboratory Animals of Peking University First Hospital and the Animal Ethics Committee of Peking University First Hospital (Approval No. J2026086).

*Transcriptomic and Proteomic Analysis.* Principal components analysis (PCA) was conducted using the prcomp function of the stats R package (version 4.3.1) to identify closely related samples. EdgeR R package (version 3.42.4) was employed to perform a significant analysis of expression differences. Differentially expressed genes (DEGs) were identified with a P value of <0.01 and an absolute log2 (fold change) of > 0.8. For functional annotation of the DEGs, the online tool Metascape (Version 3.5.20230501) was utilized to perform Gene Ontology (GO) and Kyoto Encyclopedia of Genes and Genomes (KEGG) pathway enrichment analysis. Gene Set Enrichment Analysis (GSEA) was done by org.Mm.eg.db R package (version 3.17.0) and the gseGO function of clusterProfiler R package (version 4.8.2). The enriched Go terms and KEGG pathways with a P value of < 0.01 were considered significant. To further understand the interactions among the proteins, DEGs were used to draw a protein-protein interaction (PPI) network using the Search Tool for Retrieval of Interacting Genes (STRING) database (version 12.0) and Cytoscape (version3.10.0). Center networks and hub genes were identified by the MCODE plugin (version 2.0.2). Degree Centrality (without weight) was calculated by CytoNCA plugin (version 2.1.6). To gain further insights into the co-expression relationship among transcriptomic and proteomic, the psych R package (version 2.3.6) was used to calculate the correlation (spearman) and correlation significance(adjusted by the False Discovery Rate method) of each pair of hub genes. Adjusted P value of < 0.01 was considered significant. All data was statistically analyzed and visualized by GraphPad Prism (version 8.0), R (version 4.3.1), and Cytoscape.

*Statistical Analysis.* All experimental procedures were performed in triplicate with independent biological replicates. Quantitative data are expressed as the mean ± standard deviation (SD). Statistical comparisons among groups were conducted using one-way analysis of variance (ANOVA), followed by Tukey's post hoc test for multi-group analysis, employing GraphPad Prism 10 software (Version 10.0.2). Significance thresholds were set at *p < 0.05, **p < 0.01, and ***p < 0.001, with asterisks indicating statistical significance relative to designated controls.

## 3 Results and discussion

### 3.1 Synthesis and characterization of PtPb@SbO_3-x_

**Fig. 1:**
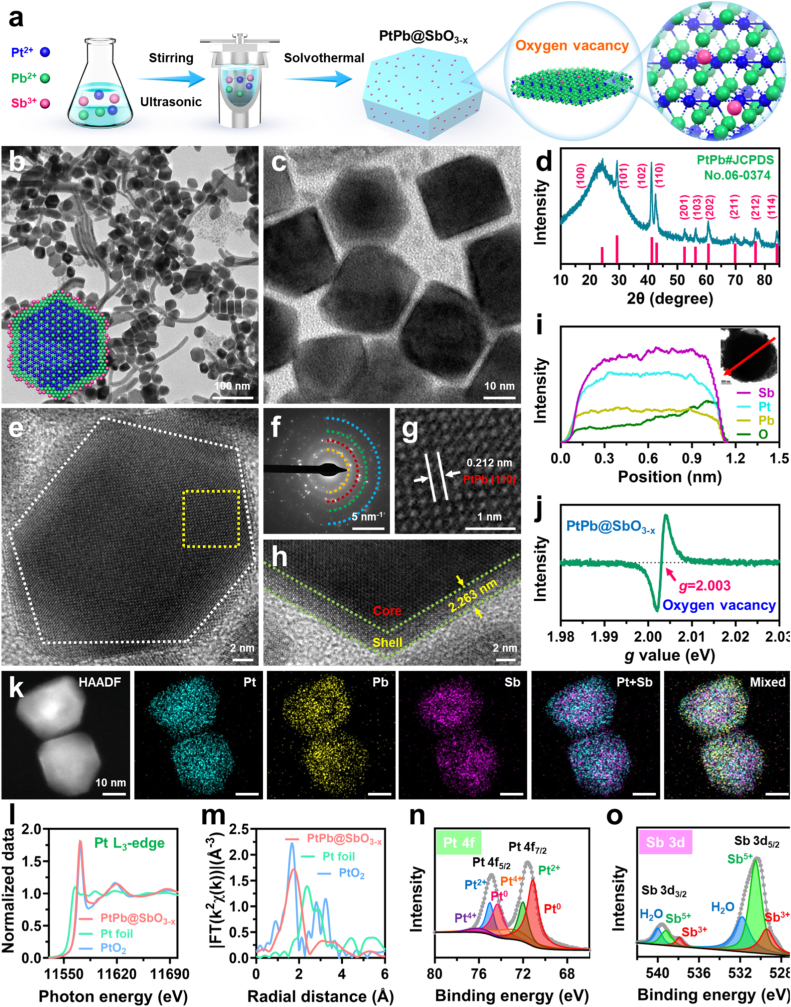
Structural characterization and morphological validation of oxygen vacancy PtPb@SbO_3-x_ nanoplatforms. (a) Schematic illustration of the synthesis procedure and structural configuration. (b) Representative TEM micrograph (the inset displays the schematic atom model from the top view), (c) HAADF-STEM image, (d) Powder X-ray diffraction (PXRD) pattern, (e) HRTEM image, and (f) Corresponding fast Fourier transform (FFT) analysis of PtPb@SbO_3-x_. (g) Magnified HRTEM view of the region outlined by the yellow dashed box in (e). (h) Atomic-resolution HRTEM image revealing crystalline lattice fringes. (i) Elemental line-scan profile across the nanostructure. (j) Electron paramagnetic resonance (EPR) spectra confirming oxygen vacancy concentration. (k) HAADF-STEM image with superimposed elemental distribution maps. (l) Pt L_3_-edge X-ray absorption near-edge structure (XANES) spectra and (m) Corresponding Fourier-transformed extended X-ray absorption fine structure (FT-EXAFS) profiles. High-resolution XPS spectra of (n) Pt 4f and (o) Sb 3d core levels in PtPb@SbO_3-x_.

Defect-rich PtPb@SbO_3-x_ nanozymes were successfully synthesized using a wet-chemical method [20], employing Pt(acac)_2_, Pb(acac)_2_, and Sb(acac)_3_ as precursors, along with L-ascorbic acid (AA) as a reducing agent and oleylamine (OAm)/1-octadecene (ODE) as solvents (Fig. 1a). The structure and morphology of PtPb@SbO_3-x_ were analyzed. The nanoparticles that were synthesized were found to have a uniform dispersion with hexagonal and square characteristics, as seen from the transmission electron microscopy (TEM) and high-angle annular dark-field scanning transmission electron microscopy(HAADF-STEM) images shown in Fig. 1b. As shown in Fig. 1c, the statistical analysis of TEM micrographs obtained from multiple fields of view revealed that the product predominantly exhibited a particulate morphology, characterized by hexagonal and square features, with a minor fraction of linear nanostructures (<10%). The linear by-products were effectively eliminated through differential centrifugation, thereby ensuring minimal interference with subsequent catalytic and biological evaluations. The average diameter of the particles was measured at 20.5 ± 0.5 nm. PXRD patterns (Fig. 1d) confirmed the PtPb core was not altered due to the introduction of surface Sb species. The hexagonal intermetallic phase structure remained unchanged. The characteristic diffraction peaks at 29.2°, 41.1°, 42.5°, 52.3°, 56.3°, 60.7°, 76.9°, and 84.1° correspond to the (101), (102), (110), (201), (103), (202), (211), (212), and (114) planes of intermetallic PtPb (JCPDS 06-0374) [21], respectively. High resolution TEM (HRTEM) imaging (Fig. 1e) elucidates the lattice structure of hexagonal nanoparticles. Fast Fourier transform (FFT) pattern (Fig. 1f) verifies the hexagonal nature. We measured the lattice spacing of 0.214 nm, which corresponds to (110) plane of PtPb intermetallic phase (Fig. 1g). As shown in the enlarged HRTEM image (Fig. 1h), the core and edge regions clearly displayed two orientations of lattice, confirming the core-shell structure. Energy-dispersive X-ray spectroscopy (EDS) line scanning (Fig. 1i) revealed an alternating distribution of Pt, Pb, and Sb and their elemental content followed an order of Sb > Pt > Pb. The elemental composition and chemical structure of PtPb@SbO_3-x_ were additionally verified using transmission electron microscopy-energy dispersive X-ray spectroscopy (TEM-EDS) and Raman spectroscopy (Figs. S1 and S2). EPR spectroscopy (Fig. 1j) exhibited a signal at *g* = 2.003 due to the oxygen vacancies present in high density. The elemental mapping obtained from high-angle annular dark field scanning transmission electron microscopy-energy dispersive X-ray spectroscopy (HAADF-STEM-EDS) shows that the as-obtained sample has a core-shell structure. The Sb was mostly distributed on the surface as shown in Fig. 1k and S3. Collectively, these results validate the successful fabrication of a composite nanozyme comprising a PtPb intermetallic core, a thin Pt shell, and surface Sb species. To investigate the electronic and coordination states of surface Pt, X-ray absorption near-edge structure (XANES) and extended X-ray absorption fine structure (EXAFS) analyses were performed [22]. The white-line intensity of Pt in PtPb@SbO_3-x_ (Fig. 1l) significantly exceeded that of Pt foil and closely matched that of PtO_2_, suggesting a predominant oxidized Pt state. Fourier-transformed k^2^-weighted EXAFS spectra (Fig. 1m–S4 and Table S1) revealed the absence of Pt-Pt metallic bonds, while the emergence of Pt-O coordination peaks with a positive shift (ΔR = 0.13 Å) and reduced intensity compared to PtO_2_ implied decreased Pt-O coordination numbers and the presence of oxygen vacancies. Wavelet transform (WT) analysis (Fig. S5) further validated the dominance of Pt-O coordination. X-ray photoelectron spectroscopy (XPS) was employed to analyze surface compositions and chemical states (Fig. S6a). The Pt 4f spectrum (Fig. 1n) exhibited three distinct components: metallic Pt^0^ (71.6 eV), Pt^2+^ (72.8 eV), and Pt^4+^ (74.2 eV) [23]. The Pb 4f spectrum (Fig. S6b) revealed peaks corresponding to Pb^0^ (136.9 eV) and Pb^2+^ (138.4 eV). The Sb 3d spectrum (Fig. 1o) indicated the presence of mixed oxidation states of Sb^3+^ (539.6 eV) and Sb^5+^ (540.8 eV). The C 1s spectrum (Fig. S6c) showed contributions from C-C (284.6 eV), C=O (285.7 eV), and C-O (288.0 eV). The O 1s spectrum (Fig. S6d) showed contributions from the surface hydroxyl groups (C-OH, 531.2 eV) and chemisorbed water (532.7 eV). The full characterization provides a structural basis for an understanding of the unique defect-rich surface structure and electronic properties of PtPb@SbO_3-x_, as well as the remarkable performance of this material as catalyst.

### 3.2 Photothermal and nanozyme properties of PtPb@SbO_3-x_

**Fig. 2:**
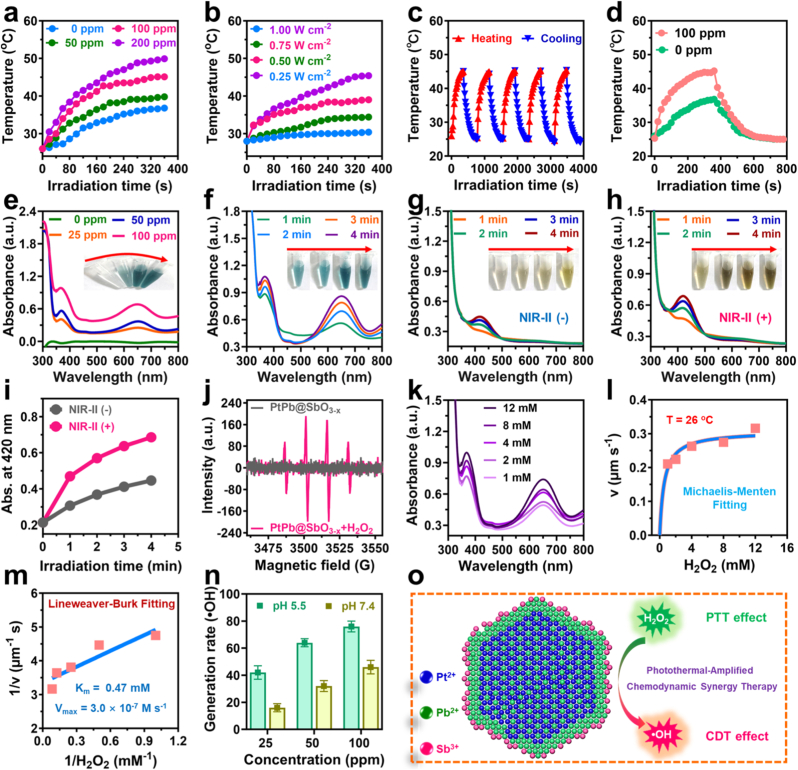
**Photothermal performance and POD-mimetic activities of PtPb@SbO_3-x_.** (a) Temperature elevation profiles of PtPb@SbO_3-x_ solutions (0-200 ppm) under 1064 nm laser irradiation (1.0 W cm^−2^). (b) Photothermal conversion efficiency at varied laser power densities (0.25-1.0 W cm^−2^) for 100 ppm nanozyme solution. (c) Cyclic photothermal stability assessment through five on/off irradiation cycles (1.0 W cm^−2^). (d) Temperature-time curves for heating/cooling phase analysis. (e) Concentration- and (f) time-dependent absorbance variations of TMB oxidation mediated by POD-mimetic activity. Temporal absorbance evolution of OPD oxidation (g) with and (h) without NIR-II irradiation, with insets showing corresponding solution coloration. (i) Comparative enhancement of POD-mimetic activity under NIR-II stimulation. (j) EPR spectra of DMPO-•OH adducts after 5 min reaction with/without H_2_O_2_ (0.1 mM). (k) H_2_O_2_ concentration-dependent TMB oxidation kinetics at 26°C. (l) Michaelis-Menten kinetics and (m) corresponding Lineweaver-Burk plot for enzymatic activity quantification. (n) pH-modulated •OH generation rates under physiological (pH 7.4) and acidic (pH 5.5) conditions. (o) Proposed catalytic mechanism of the PtPb@SbO_3-x_ nanozyme system.

To verify the rationality of the material system design, the photothermal conversion performance and biocatalytic activity of PtPb@SbO_3-x_ were systematically evaluated. Based on the composition characteristics of this material, we expected this material could behave like POD to produce highly toxic •OH via the degradation of H_2_O_2_ [24]. To confirm our expectation, we systematically characterized the performance of the nanomaterial by following their thermogenic and radical generation processes. The UV-vis-NIR spectra (Fig. S7) showed that PtPb@SbO_3-x_ has a strong absorption of light in the NIR-II biowindow (1000-1350 nm), which provides an optical basis for effective photothermal conversion under laser excitation at 1064 nm. Interestingly, it is obvious that as the concentration of the material was increased, its absorbance at 1064 nm (Fig. S8) increased. This is evidence of its photothermal conversion ability. As shown in quantitative temperature measurements (Fig. 2a), the solution of 100 ppm material reached 45.2°C in 5 min upon 1064 nm irradiation, showing features of moderate thermal effect. Power-dependent experiments (Fig. 2b) confirmed the correlation of temperature growth with laser power and stability for five heating-cooling cycles (Fig. 2c), indicating high stability as a PTT agent. Meanwhile, The hydrodynamic diameter of PtPb@SbO_3-x_ nanoparticles decreased from 282.9 nm at t = 24 h to 222.2 nm after 48 h in PBS buffer (pH 7.4), while the zeta potential changed from −8.22 to −18.00 mV (Fig. S9). This decrease in particle size can be attributed to the slight dissociation of the weak aggregates originally present, which occurs due to an enhanced electrostatic repulsion that develops gradually. Conversely, the significant increase in negative zeta potential can be primarily attributed to the hydration of surface oxygen vacancies on the SbO_3-x_ shell and the deprotonation of hydroxyl groups. These results suggest that the nanozyme exhibits good colloidal stability in biological media, and its surface chemistry approaches equilibrium over time, which is beneficial for the biological application and sustainable catalytic activity of the nanozyme. The calculated photothermal conversion efficiency was determined to be 58.6% (Fig. 2d–S10, and Table S2), much higher than the existing reports, ensuring stringent applicability for antibacterial applications [25,26]. The POD-mimetic enzymatic activities were confirmed using TMB and OPD chromogenic assays. The prominent absorption peaks (oxTMB@652 nm and oxOPD@417 nm) which are exhibited by the materials intensified with increasing concentration of the materials as well with the reaction times (Fig. 2e and f). Moreover, the above was the behaviour which was expected in case of concentration and time upon dependent behaviour. Notably, the joint influence of NIR-II laser (Fig. 2g–i) was instrumental in enhancing the catalytic activity toward the process, as seen from the prominent peak intensity at 420 nm with a 24.0% increase. This indicates the photothermal effect definitely improved enzymatic activity. Electron spin resonance (ESR) spectra (Fig. 2j) provided additional evidence for substantially generated •OH signals under NIR-II irradiation, confirming their photothermal-catalytic synergistic effect. The steady-state kinetic analysis (Fig. 2k–m) conformed to the Michaelis-Menten model, providing a maximum velocity (Vmax) of 3.0 × 10^−7^ M s^−1^ and a Michaelis constant (Km) of 0.47 mM [27,28]. A low value of Km designates high affinity to the substrate H_2_O_2_, while a high Vmax value indicates high catalytic efficiency compared with other nanozymes (Table S3). Salicylic acid (SA) trapping assays (Fig. 2n) showed that, compared with neutral (pH 7.4) environments, •OH generation was increased by 2.3 folds in acidic (pH 5.5) environments, implying SA's efficacy in acidic infection microenvironments. Multifaceted experiments demonstrate that PtPb@SbO_3-x_ shows high efficiency in photothermal conversion and cyclic stability. It also shows notable POD-mimetic activity and enhanced catalytic effect owing to photothermal stimulation (Fig. 2o). These results will provide valuable experimental evidence towards the development of any nanoagents with antibacterial properties.

### 3.3 The density functional theory (DFT) calculation of PtPb@SbO_3-x_

**Fig. 3:**
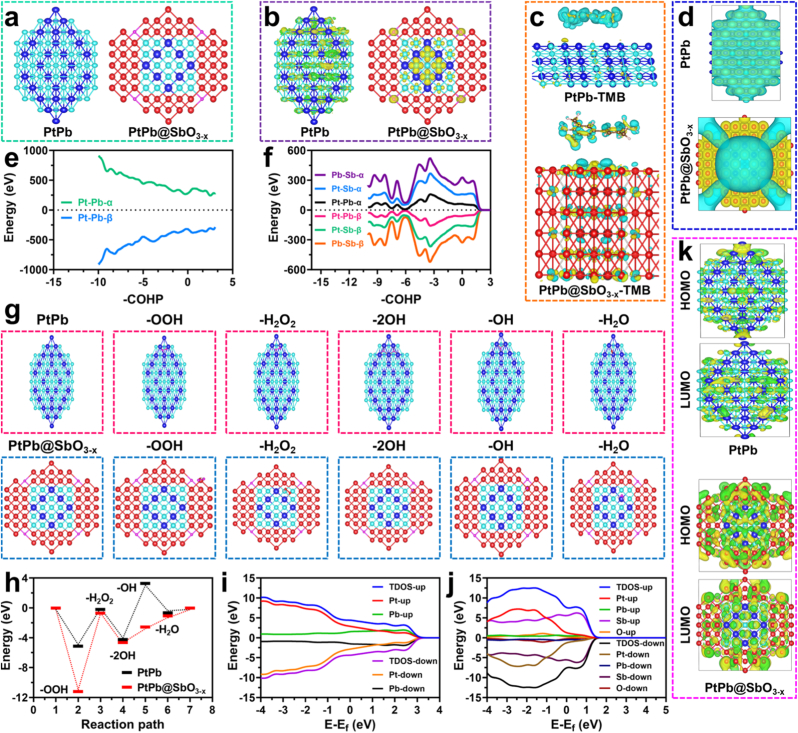
**Density functional theory (DFT) mechanistic investigations of PtPb@SbO_3-x_ nanozyme.** (a) Optimized geometries and (b) spin-polarized charge density distributions (yellow: spin-up electrons; green: spin-down electrons) for PtPb and PtPb@SbO_3-x_. (c) Differential charge density profiles of PtPb-TMB and PtPb@SbO_3-x_-TMB complexes. (d) Surface electrostatic potential mappings (blue: negative charge; yellow: positive charge) for PtPb and PtPb@SbO_3-x_. Crystal orbital Hamilton population (COHP) analyses for (e) PtPb and (f) PtPb@SbO_3-x_. (g) Optimized adsorption configurations and catalytic decomposition pathways of H_2_O_2_ at Pt active sites. (h) Comparative Gibbs free energy profiles for •OH generation from H_2_O_2_ decomposition. Projected density of states (pDOS) for (i) PtPb and (j) PtPb@SbO_3-x_. (k) Frontier molecular orbital energy levels with HOMO-LUMO interfacial charge distribution diagrams. All calculations were performed using DFT with the CAM-B3LYP range-separated hybrid functional and 6-31G(d) basis set.

This study performed DFT calculations to elucidate the interaction mechanism between SbO_3-x_ and PtPb, thereby explaining the remarkable POD-mimetic catalytic activity of PtPb@SbO_3-x_ and its impact on the electronic structure [29]. The models of PtPb and PtPb@SbO_3-x_ were initially optimized, considering the atomic structure of a face-centered cubic (fcc) lattice (Fig. 3a). As shown in Fig. 3b, the charge density analysis of spin indicates that spin-polarized charges are localized at the Pt and Sb sites. The neighboring O atoms of Pt and Sb exhibit enhanced spin polarization due to d-p hopping, which is crucial for improving both reactant adsorption and electron transfer, thereby providing a theoretical foundation for the observed enhanced catalytic activity. The adsorption energy (Eads) analysis for the TMB substrate reveals that PtPb@SbO_3-x_ interacts more strongly with TMB (−0.668 eV) as compared to pristine PtPb (−0.601 eV) which aligns with the experimental Km values (Fig. 3c and S11). According to the charge density difference analysis depicted in Fig. 3d, a significant distinction exists in the charge distributions at the interface; specifically, the PtPb substrate accumulates positive charges (indicated by the blue area), while the SbO_3-x_ accumulates negative charges (indicated by the yellow area). This observation suggests strong charge transfer, predominantly from SbO_3-x_ to PtPb, thereby confirming robust interfacial interactions between the two materials. The work function calculation indicates that PtPb@SbO_3-x_ has a work function of 4.79 eV, which is higher than that of PtPb at 4.63 eV to promoting interfacial electron transfer (Fig. S12). As illustrated in Fig. 3e and f, the PCOHP analysis reveals the contributions of the Pt and Sb sites in H_2_O_2_ adsorption. In clean PtPb, the Pt-Pb atomic pairs contributed equivalently to H_2_O_2_ adsorption through α/β spin states. In contrast, for PtPb@SbO_3-x_, similar electronic distributions in the antibonding regions of Pt-Pb, Pt-Sb, and Pb-Sb pairs are observed for both spin states, indicating effective valence alloying at the interface. According to the reaction pathway analysis (Fig. 3g), H_2_O_2_ in PtPb@SbO_3-x_ is preferentially activated via the Sb site to generate -OOH* intermediates, followed by O-O bond breaking, which produces -2OH* reactive species. The DFT calculated difference in the adsorption energies of the two sites (PtPb: −0.601 eV, PtPb@SbO_3-x_: −0.668 eV, Fig. 3h) demonstrates that the composite structure exhibits superior H_2_O_2_ adsorption and activation capacity, consistent with the enhanced catalytic performance observed experimentally. The band structure analysis (Fig. 3i and j, and S13) reveals considerable hybridization among the Pt-d, Pb-p, Sb-d, and O-p orbitals near the Fermi level of PtPb@SbO_3-x_. The electronic structure shift induced by SbO_3-x_ is evidenced by an upward shift of the Fermi level, which is 2.58 eV for PtPb@SbO_3-x_ compared to 0.01 eV for PtPb. Furthermore, DFT calculations of the HOMO-LUMO energy gap confirm a reduced bandgap for PtPb@SbO_3-x_ (0.0109 eV) compared to PtPb (0.0427 eV) which is beneficial for the electron excitation and interfacial charge transfer thus enhancing the kinetics of the catalytic reactions (Fig. 3k). In summary, the DFT calculations provide a detailed understanding of the factors contributing to the excellent catalytic efficiency, including the reconstruction of the electronic structure of PtPb through interfacial charge transfer, enhancement of surface spin polarization, optimization of the adsorption strength of reaction intermediates, and a synergistic enhancement of POD-mimetic activity. This study establishes a significant theoretical framework for the rational design of high-performance nanozymes.

### 3.4 Metabolomics reveals antibacterial and antibiofilm of PtPb@SbO_3-x_

**Fig. 4:**
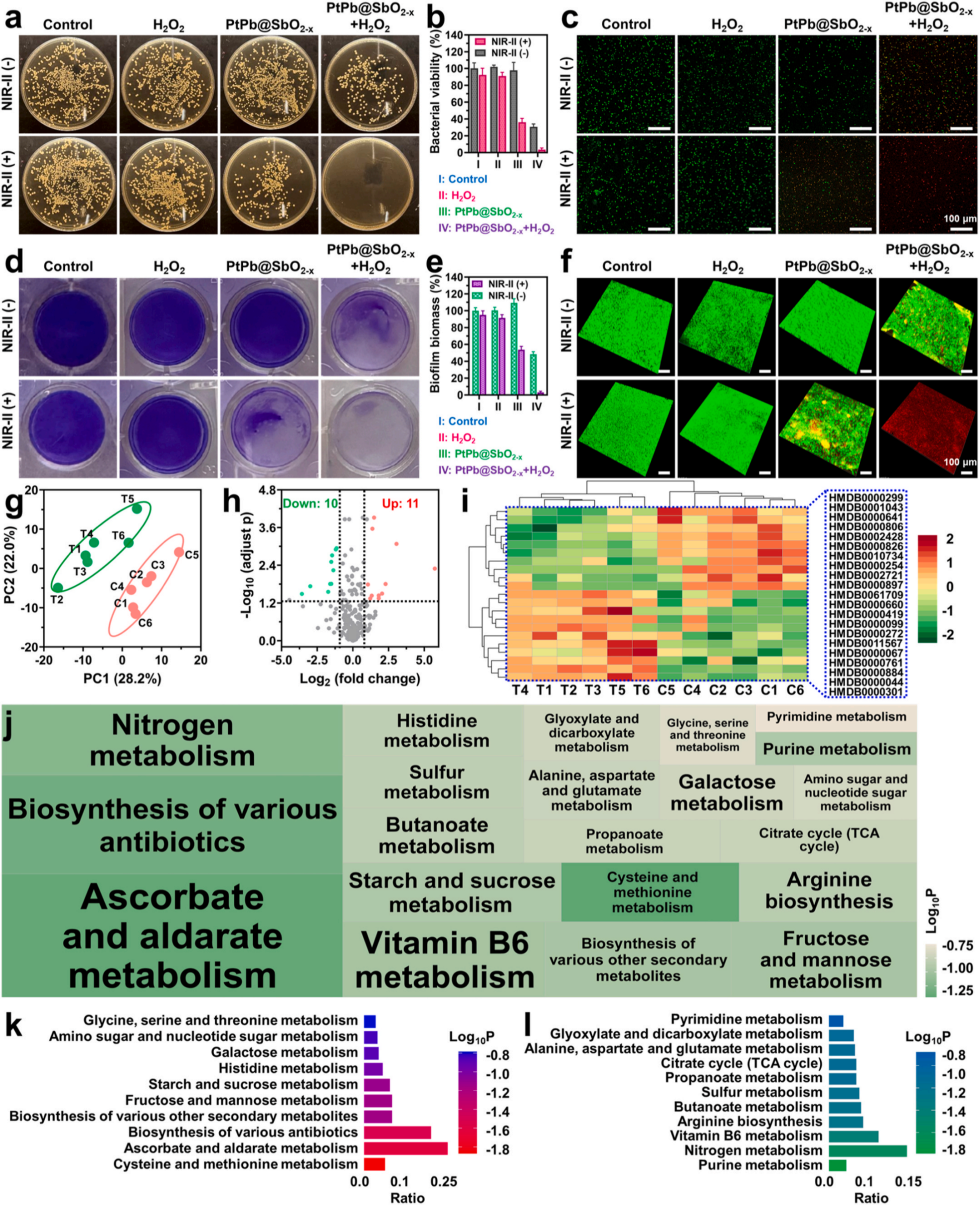
**Antibacterial and antibiofilm efficacy of PtPb@SbO_3-x_ nanozymes with mechanistic metabolomic insights.** (a) Digital photographs and (b) corresponding quantitative survival rates of MRSA colonies under diverse treatment conditions (mean ± SD, n = 3). (c) Confocal laser scanning microscopy (CLSM) images of live/dead-stained MRSA post-treatment (green: viable SYTO 9-stained cells; red: membrane-compromised PI-stained cells). (d) Optical micrographs and (e) quantitative crystal violet staining analysis of MRSA biofilms after exposure to PtPb@SbO_3-x_ under varying experimental conditions (I: Control, II: H_2_O_2_, III: PtPb@SbO_3-x_, IV: PtPb@SbO_3-x_ + H_2_O_2_; mean ± SD, n = 3). (f) Three-dimensional CLSM reconstruction of residual biofilms, illustrating live/dead bacterial distribution. Metabolomic profiling of antibacterial mechanisms: (g) Principal Component Analysis (PCA) score plot distinguishing metabolic profiles between control (pink) and PtPb@SbO_3-x_-treated (green) groups. (h) Volcano plot highlighting differentially regulated metabolites (pink: upregulated; green: downregulated; thresholds: Log_2_(fold change) > 0.8, P < 0.01). (i) Heatmap visualizing hierarchical clustering of significantly altered metabolites. (j) KEGG orthology (KO) enrichment treemap, with color intensity reflecting pathway significance (P-value magnitude). KEGG pathway enrichment analysis of (k) upregulated and (l) downregulated metabolite-associated pathways.

Due to the unique biocatalytic action, photothermal activity, and biocompatibility of PtPb@SbO_3-x_, this study systematically evaluates its antibacterial capacity against MRSA and its biofilm eradication capabilities. The synergistic effects of CDT and PTT exhibited by this material demonstrate an excellent bactericidal effect and biofilm removal. The antibacterial activities of free PtPb@SbO_3-x_ showed minimal activity under standard colony counting assays compared to PBS control experiments (Fig. 4a and b and S14). However, its inhibition cagainst MRSA significantly increases under NIR-II irradiation. Notably, H_2_O_2_ induces a 74.8% inactivation rate of MRSA by PtPb@SbO_3-x_ through effective CDT. The combination of NIR-II irradiation with CDT (CDT/PTT group) achieves a bacterial eradication efficiency exceeding 99.5%, attributed to the NIR-II-enhanced catalytic activity of PtPb@SbO_3-x_. Results from SYTO-9/PI double-staining fluorescence microscopy (Fig. 4c and S15, confirm that MRSA in the PBS and free PtPb@SbO_3-x_ groups predominantly emit green fluorescence, indicative of live bacteria. In contrast, red fluorescence, indicative of dead bacteria, significantly increases in reactions containing either CDT or NIR-II alone. All bacteria are stained red, indicating membrane damage caused by incessant ROS formation due to CDT/PTT treatment. Observations using scanning electron microscopy (SEM) (Fig. S16) confirm that the membranes in the CDT/PTT group have severely shrunk and collapsed in structure, suggesting that ROS are responsible for this damage. Regarding the clinical challenge posed by biofilms, crystal violet and fluorescence staining (Fig. 4d–f) indicate that PtPb@SbO_3-x_ alone does not significantly affect MRSA biofilm formation. However, both biofilm biomass and bacterial viability were effectively reduced under H_2_O_2_ or NIR-II stimulation. Most importantly, the CDT/PTT group resulted in significant biofilm disintegration, as confirmed by 3D confocal laser scanning microscopy (CLSM) images. Thse images revealed a loose biofilm structure stained red (indicating dead bacteria) while the control biofilm remained intact and stained green (indicating live bacteria). This synergy is likely attributed to the photothermal enhancement of nanomaterial internalization and an increase in ROS generation deep within the biofilm. Through catalytic ROS production and the photothermal effect, PtPb@SbO_3-x_ can effectively eliminate MRSA and its biofilms via CDT/PTT synergy. Specifically, under NIR-II irradiation, the first mechanism generates increased ROS while the second enhances bacterial membrane permeability and material uptake. This synergistic strategy offers a novel nanomaterial-based solution for the clinical treatment of drug-resistant infections and biofilm-associated diseases. To elucidate the antibacterial mechanism of PtPb@SbO_3-x_, bacterial metabolomics was employed to analyze the metabolic network of MRSA [30]. Metabolomic profiling identified 268 metabolites (Figs. S17 and S18, Table S4). Principal component analysis (PCA) revealed a distinct separation between treated and control groups (Fig. 4g and S19), indicating significant metabolic perturbation. Differential analysis identified 21 significantly altered metabolites (Fig. 4h and i; Table S5). The KEGG pathway enrichment analysis identified several key pathways, including “ascorbate and aldarate metabolism”, “biosynthesis of various antibiotics”, and “nitrogen metabolism” (Fig. 4j). Upregulated metabolites (11) — isoheptadecanoic acid, D-fructose, 3-epicholic acid, L-cystathionine, O-phosphoserine, 1-monoolein, cholesterol, lithocholic acid, 5-methyluridine, ascorbic acid, and urocanic acid — were significantly enriched in the “cysteine and methionine metabolism”, “ascorbate and aldarate metabolism”, and “antibiotic biosynthesis” pathways (Fig. 4k). The elevated levels of ascorbic acid suggest a state of oxidative stress, while the activation of antibiotic biosynthesis may indicate responses to bacterial resistance. Conversely, downregulated metabolites (10) — xanthosine, arachidonic acid, L-glutamine, myristic acid, terephthalic acid, pentadecanoic acid, 3-hydroxypalmitic acid, succinic acid, 1-methylinosine, and 7-methylguanine — were associated with the “purine metabolism”, “nitrogen metabolism”, and “vitamin B6 metabolism” pathways (Fig. 4l). The reduction of purine intermediates likely inhibits DNA/RNA synthesis, while the disruption of nitrogen metabolism impairs energy and amino acid synthesis. These metabolic shifts indicate two antibacterial pathways: (1) oxidative damage via ROS burst and ascorbate pathway upregulation, and (2) metabolic suppression through impaired nucleotide synthesis, coenzyme dysfunction, and nitrogen metabolism dysregulation. This multi-target metabolic reprogramming mechanism underlies the high antibacterial efficacy of PtPb@SbO_3-x_.

### 3.5 Treatment of MRSA infectious pneumonia

**Fig. 5:**
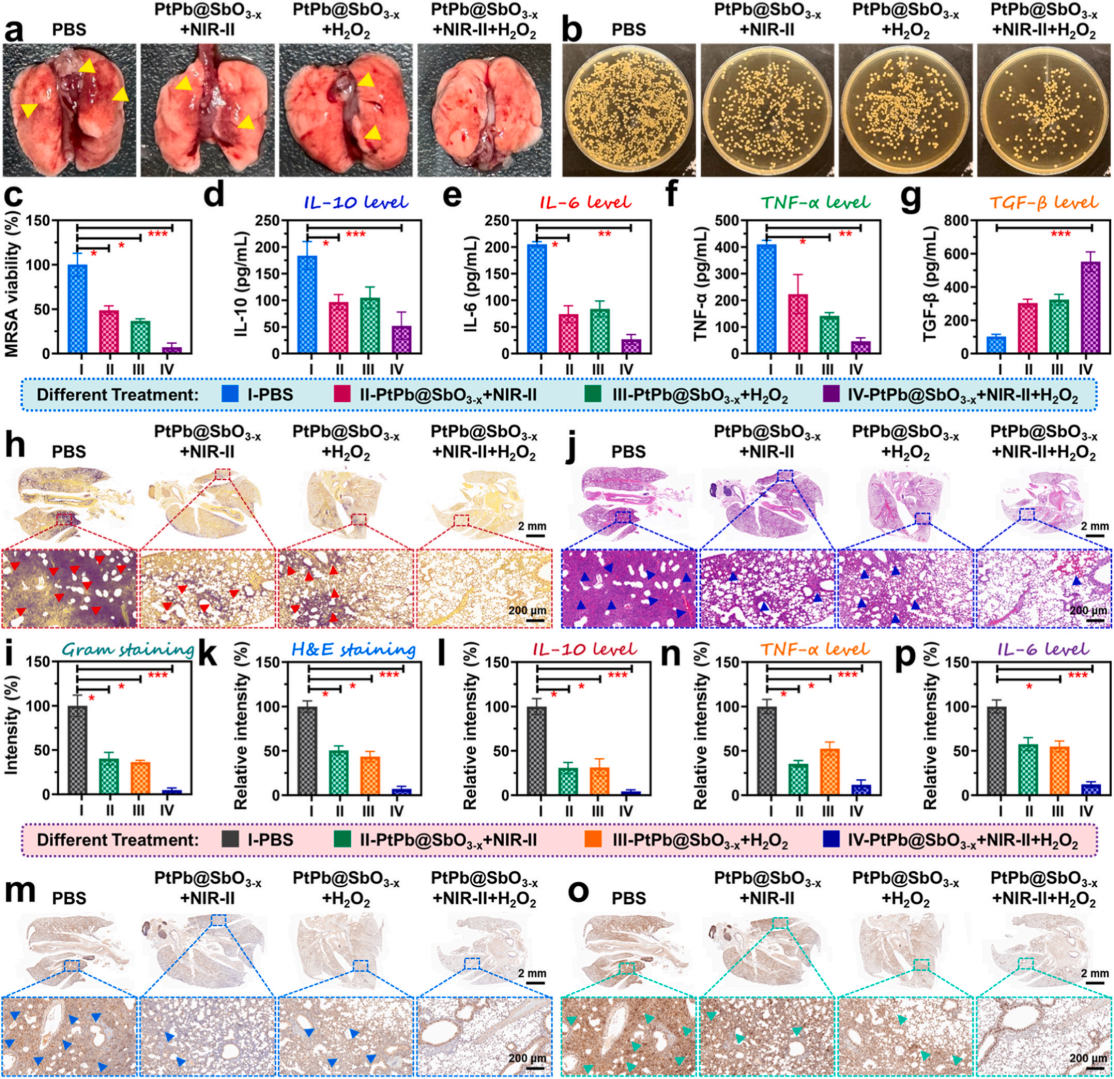
***In vivo* therapeutic evaluation of PtPb@SbO_3-x_ against MRSA-induced bacterial pneumonia.** (a) Macroscopic morphology of excised lung tissues from different treatment groups. (b) Representative images illustrating biofilm formation in infected lungs across experimental conditions. (c) Quantitative analysis of bacterial burden in pulmonary tissues. Cytokine profiling in BALF showing concentration variations of (d) IL-10, (e) IL-6, (f) TNF-α, and (g) TGF-β among treatment cohorts. Histopathological characterization: (h) Gram-stained sections with quantitative analysis of residual MRSA colonization (i), and (j) H&E-stained sections with quantitative assessment of inflammatory infiltration (k). Immunohistochemical evaluation of proinflammatory cytokine expression: (l) IL-10 corresponding densitometric quantification; (m) TNF-α-positive signals and (n) corresponding densitometric quantification; (o) IL-6-positive signals and (p) statistical analysis of staining intensity. Data represent mean ± SD (n = 3), biologically independent animals randomly selected from the original 5 per group. Statistical significance: *p < 0.05, **p < 0.01, ***p < 0.001.

Based on preliminary verification of the antibacterial activity of PtPb@SbO_3-x_ *in vitro*, to further constructed an MRSA-induced deep pneumonia infection model and systematically evaluated its *in vivo* therapeutic performance [31]. Through a respiratory nebulization administration strategy, we implemented various interventions: PBS control, PtPb@SbO_3-x_ + CDT, PtPb@SbO_3-x_ + PTT, and PtPb@SbO_3-x_ + CDT + PTT. After 24 h of ex vivo lung tissue analysis (Fig. 5a), the control group exhibited significant congestion and swelling of lung lobes, while the synergistic treatment group effectively maintained the normal physiological state of lung tissue. By quantitatively evaluating the antibacterial efficacy through colony counting of lung tissue homogenate on agar plates (Fig. 5b and c), we observed that the colony count in the synergistic treatment group was significantly reduced compared to the control group, demonstrating greater efficacy than either single treatment mode. To verify the changes in lung tissue inflammation, we utilized ELISA to detect the levels of key inflammatory factors in BALF. As shown in Fig. 5d–g, the concentrations of interleukin-10 (IL-10), IL-6, and TNF-α in the synergistic treatment group were significantly lower than those in the control group (P < 0.001), whereas the PBS or TNF-α alone-treated PtPb@SbO_3-x_ group maintained a high level of inflammation. Notably, IL-10 exhibits a context-dependent dual role in infectious inflammation. In cases of uncontrolled acute bacterial pneumonia, markedly elevated levels of IL-10 in BALF are commonly associated with compensatory immunosuppression, impaired pathogen clearance, and poor prognosis. However, in our therapeutic setting, the significant reduction of IL-10 in the combination therapy group (Fig. 5d) indicates an effective resolution of the infection and a consequent alleviation of excessive immunosuppressive drive, which is consistent with a favorable treatment response. The concentration of TGF-β in the collaborative treatment group was significantly higher than that in the control group (P < 0.001), indicating that the inflammatory response was effectively inhibited. This evidence strongly supports the conclusion that PtPb@SbO_3-x_ can effectively eliminate deep-seated infectious pathogens through a synergistic therapeutic mechanism, which significantly reduces lung tissue damage by modulating the inflammatory cascade. Further investigation into the antibacterial effects and inflammatory responses in lung tissue were conducted via pathological section analysis. As illustrated in Fig. 5h and i, the synergistic effects of CDT and PTT can effectively penetrate biofilm barriers and eradicate drug-resistant bacteria, closely aligning with the results of lung tissue homogenate plate counting (as indicated by the red arrow). Notably, H&E staining revealed a reduction in neutrophil infiltration within the synergistic treatment group (Fig. 5j and k) and a significant restoration of tissue structural integrity (as indicated by the blue arrows). To analyze the host immune response throughout the treatment process, multiple indicators were assessed through immunohistochemistry to evaluate the dynamic changes in inflammatory factors. As illustrated in Figure S20a and 5l-5p, synergistic therapy significantly downregulated the expression of pro-inflammatory cytokines, including IL-10 (reduced by 95%), TNF-α (reduced by 90%), and IL-6 (reduced by 88%) (p < 0.001), while simultaneously upregulating the growth factor VEGF (Fig. S20b). Immunohistochemistry further elucidated key regulatory mechanisms: the expression of M1 macrophage markers was inhibited, while the expression of M2 macrophage markers was activated, indicating that the material reshapes the inflammatory microenvironment by regulating the polarization of macrophage phenotypes (from M1 to M2). Consequently, it was confirmed through the MRSA-induced pneumonia model that PtPb@SbO_3-x_ represents a novel strategy with the translational potential for treating deep-seated tissue infections through the cascade regulatory effects of antibacterial, anti-inflammatory, and healing promotion.

**Fig. 6:**
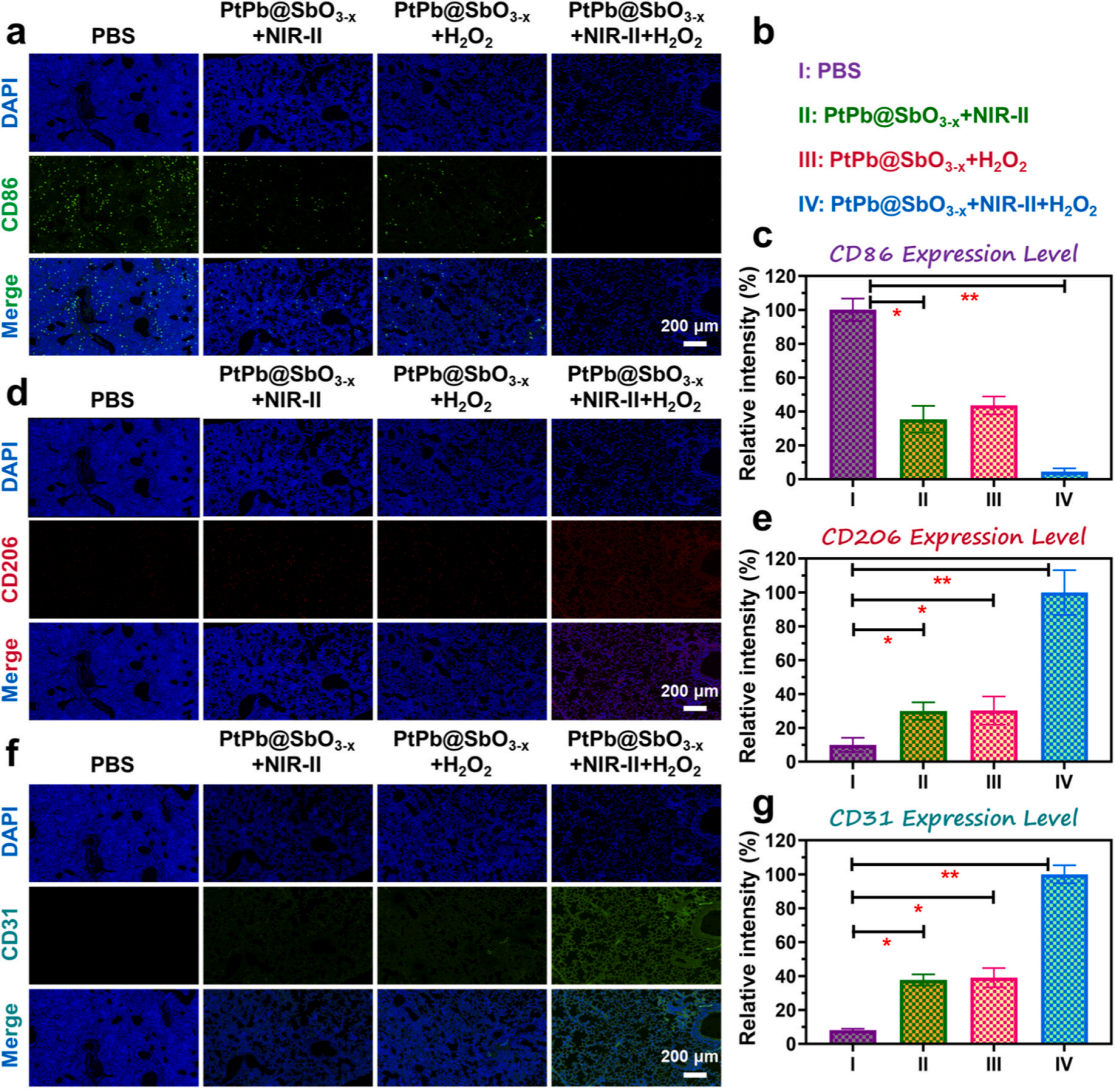
**Immunofluorescence histochemistry and quantitative analysis of pulmonary biomarkers.** (a) Representative immunofluorescence micrographs demonstrating CD86 M1-polarized macrophage infiltration in lung tissue sections across treatment groups. (b) Schematic workflow of the experimental therapeutic protocol. (c) Quantitative fluorescence intensity analysis of CD86 M1 macrophage density in pulmonary parenchyma. (d) Immunofluorescence visualization of CD206 M2 macrophage spatial distribution patterns. (e) Comparative quantification of CD206 M2 macrophage subpopulations under different treatment conditions. (f) Vascular endothelial marker CD31 expression profiles visualized through dual-channel immunofluorescence imaging. (g) Quantitative evaluation of CD31 vascular endothelial cell density as an angiogenesis indicator. Data represent mean ± SD (n = 3), biologically independent animals randomly selected from the original 5 per group. Statistical significance was determined by one-way ANOVA with Tukey's post hoc multiple comparison tests (p < 0.05, *p < 0.01, **p < 0.001).

The repair of pulmonary tissue following infection is critically influenced by the dynamic balance between M1 (pro-inflammatory) and M2 (anti-inflammatory) macrophages. To evaluate the effect of PtPb@SbO_3-x_ combined with NIR-II irradiation on macrophage phenotypic polarization, we performed immunofluorescence staining on lung tissue sections post-treatment, utilizing CD86 (an M1 marker) and CD206 (an M2 marker) as indicators of inflammatory status. As illustrated in Fig. 6a–e, the PtPb@SbO_3-x_ + H_2_O_2_ + NIR-II group exhibited a significant decrease in CD86 expression and a marked increase in CD206 expression compared to the other groups, indicating a promoted transition from M1 to M2 phenotype. M2 macrophages secrete various of angiogenic factors and anti-inflammatory mediators that facilitate tissue regeneration. The results indicate that under NIR-II irradiation, PtPb@SbO_3-x_ results in macrophage polarization towards a reparative phenotype, ultimately alleviating inflammation and restoring lung tissue structure. Furthermore, CD31 immunofluorescence staining was employed to investigate vascularization in the wound area. The findings revealed a significant enhancement in CD31 signals within the PtPb@SbO_3-x_ + H_2_O_2_ + NIR-II group (Fig. 6f). The mean fluorescence intensity of CD31 across the PBS, PtPb@SbO_3-x_ + NIR-II, PtPb@SbO_3-x_ + H_2_O_2_, and PtPb@SbO_3-x_ + H_2_O_2_ + NIR-II groups exhibited an ascending trend (Fig. 6g), thereby confirming the synergistic effect of the combined treatment on vascular remodeling. In summary, PtPb@SbO_3-x_ under NIR-II photoactivation in the presence of H_2_O_2_ demonstrates a remarkable ability to modulate the immune microenvironment and stimulate angiogenesis, leading to significant improvement in tissue repair and functional recovery at the infection site.

### 3.6 Combined Transcriptomic and Proteomic Analysis of PtPb@SbO_3-x_

**Fig. 7:**
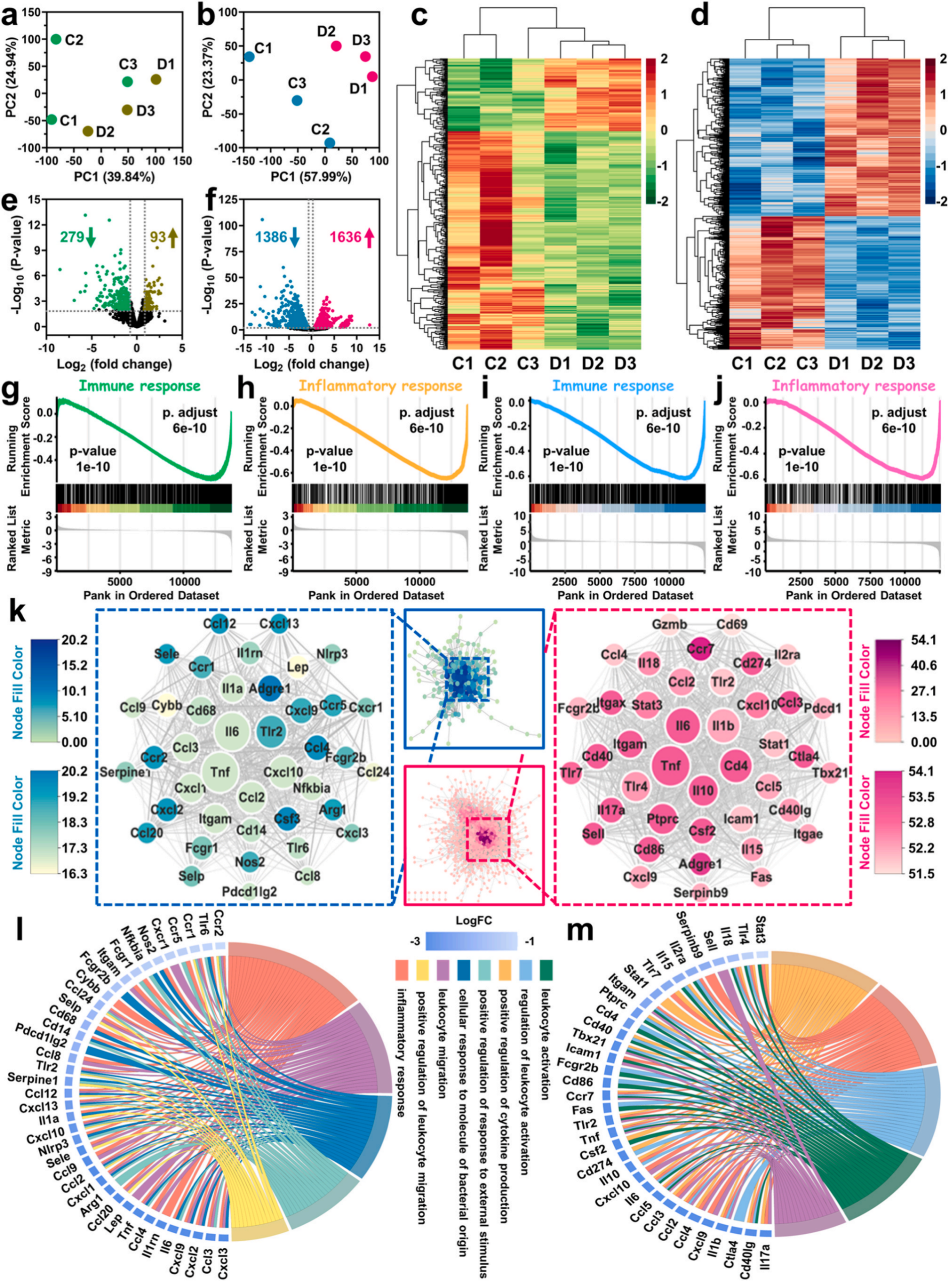
**Illustrates a comprehensive multi-omics analysis of murine lung tissue before and after treatment with PtPb@SbO_3-x_ nanozymes.** Principal component analysis (PCA) of (a) transcriptomic and (b) proteomic profiles. Heatmap visualization of differentially expressed (DE) genes in (c) transcriptomic and (d) proteomic analyses (Red indicates relatively high expression, while green and blue indicate relatively low expression). Volcano plots comparing gene expression patterns between control and PtPb@SbO_3-x_-treated groups in (e) transcriptomic and (f) proteomic analyses (black: non-significant changes; aquamarine/dark cyan: down-regulated genes; olive/deep pink: up-regulated genes). Gene Set Enrichment Analysis (GSEA) of significantly enriched Gene Ontology biological process from (g, h) transcriptomic and (i, j) proteomic data. (k) Protein-protein interaction network analysis of down-regulated DE genes (blue background: transcriptomic data; deep pink background: proteomic data). Transcriptomics and proteomics use node scores cutoff equal to 0.2 and 0.05 to identify central network diagrams, respectively. Circos plots displaying TOP5 BP terms enrichment of hub genes in (l) transcriptomic and (m) proteomic center PPI network.

**Fig. 8:**
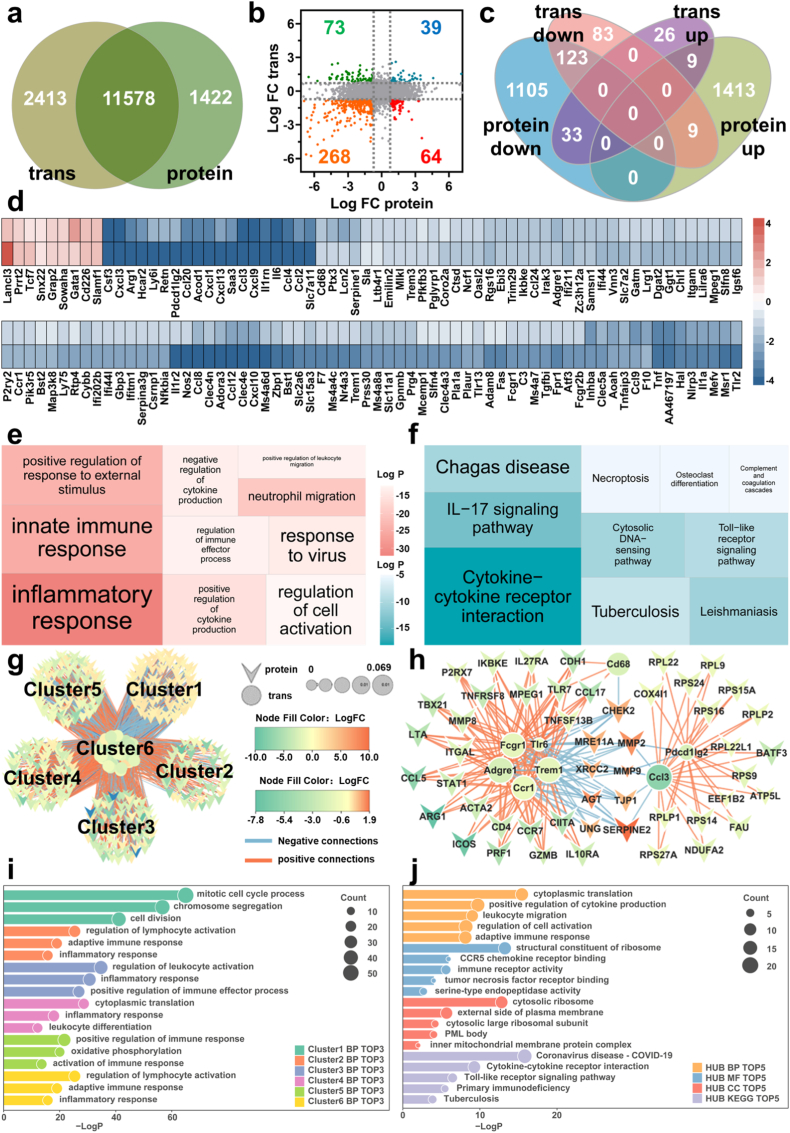
**Integrative analysis of transcriptomic and proteomic profiles in pulmonary.** (a) Venn diagram representing the quantity relationship between 13,000 identified proteins and 13,991 identified protein-coding transcripts. Green and brown sectors represent 13,000 identified proteins and 13,991 protein-coding transcripts, respectively. (b) Correlation scatter plot of gene expression abundance between proteomic and transcriptomic measurements (n = 11,578 overlapping genes). Color-coded subgroups indicate distinct expression patterns: blue (n = 39), green (n = 73), orange (n = 268), and red (n = 64). (c) Venn diagram comparing differentially expressed genes (DEGs) between omics approaches. (d) Heatmap visualization of log_2_FC values for 131 concordantly regulated DEGs (9 upregulated, 123 downregulated) across both omics platforms. Functional annotation of concordant DEGs: (e) Treemap of top 10 enriched GO biological processes; (f) KEGG pathway enrichment map. Color intensity reflects log_10_(P value), while tile size indicates gene counts per category. (g) Integrated co-expression networks: Proteome-centered PPI clusters (Cluster 1-5, concave quadrilaterals) and transcriptome-centered network (Cluster 6, circles). Edge colors denote positive (red) or negative (blue) correlations. Node fill color represents log_2_FC magnitude, with size proportional to statistical significance. (h) Central hub network extracted from panel (g). (i) Top 3 GO biological processes enriched in each network cluster. The dot size corresponds to gene numbers, horizontal axis shows enrichment significance (-log_10_(P_value_)). (j) Comprehensive functional landscape showing enriched TOP5 GO categories (BP: biological process; MF: molecular function; CC: cellular component) and KEGG pathways for network-associated genes.

The mechanism of action for treating bacterial pneumonia using PtPb@SbO_3-x_ was systematically studied in this research through a combined transcriptome and proteome analysis strategy [32]. High-throughput omics analysis revealed a total of 17,694 mRNAs and 15,674 proteins. After excluding nearly non-expressed mRNAs and proteins, we identified 13,991 mRNAs (Fig. S21 and Table S4) and 13,000 proteins (Fig. S22 and Table S6). The principal component analysis (PCA) results for transcriptomics and proteomics are illustrated in Fig. 7a and b, respectively, where “C” denotes the control group and “D” indicates the treatment group. By applying uniform threshold of P < 0.01 and absolute log_2_ FC > 0.8, we identified differential genes and proteins. Volcano plots were utilized to analyze gene and protein expression in both transcriptomics and proteomics. Transcriptomics identified 372 differentially expressed mRNAs, including 93 upregulated and 279 downregulated genes (Fig. 7c and e). In contrast, proteomics identified 3022 differentially expressed proteins, including 1636 upregulated and 1386 downregulated proteins (Fig. 7d and f). Furthermore, gene set enrichment analysis (GSEA) of both transcriptomics and proteomics revealed that after CDT + PTT treatment, pathways related to the “immune response” and “inflammatory response” were consistently downregulated, indicating a significant improvement in inflammation during the later stages of treatment (Figs. S23, S24, and 7g-7j). Subsequently, we constructed protein-protein interaction (PPI) network diagrams for downregulated mRNAs (transcriptomics, represented by blue solid line circles) and proteins (proteomics, represented by red solid line circles) separately (Fig. 7k) and extracted their central networks (represented by blue and red dashed line circles). The hub genes common to both omics included Adgre1, Ccl2, Ccl3, Ccl4, Cxcl10, Cxcl9, Fcgr2b, Il6, Itgam, Tlr2, and Tnf. Metascape analysis was employed to functionally enrich the hub genes across the two omics (Figs. S25 and S26), and chord plots were utilized to visualize the top five biological processes (BP) results from transcriptomics (39 genes) and proteomics (35 proteins) (Fig. 7l and m), revealing that the hub genes were predominantly associated with leukocyte activation and migration (Figs. S27 and S28). To explore the relationship between transcriptomics and proteomics, we conducted a joint analysis of 11,578 genes (Fig. 8a) expressed in both omics [33]. Fig. 8b illustrates the expression of these 11,578 genes across the two datasets, with the auxiliary line indicating an absolute log_2_ FC = 0.8. Utilizing differential genes identified with P < 0.01 and absolute log_2_FC > 0.8, we obtained 283 differential mRNAs (68 upregulated and 215 downregulated) and 2692 differential proteins (1431 upregulated and 1261 downregulated). Among these, 132 genes exhibited consistent expression in both omics, while 42 genes displayed opposing expression patterns (Fig. 8c and d, and S29). As depicted in Fig. 8e and f, Gene ontology (GO), biological process (BP), and Kyoto Encyclopedia of Genes and Genomes (KEGG) functional enrichment analyses of genes exhibiting consistent expression across both omics were conducted, revealing associations with pathways such as immune response, inflammatory response, and IL-17 signaling. To investigate the co-expression relationship between transcriptomics and proteomics, we extracted central networks with PPI network cluster scores greater than 10 for differentially expressed genes and visualized the correlations between hub gene pairs (Fig. 8g). The GO BP functional enrichment results for each cluster are presented in Fig. 8i. Cluster 1, primarily regulated above, was associated with the “mitotic cell cycle process”, “chromosome segregation”, and “cell division”, while clusters 2 to 6, primarily regulated below, were linked to immune inflammation-related functions (e.g., inflammatory response). Furthermore, we re-extracted the central network of the co-expression network (Fig. 8h) and enriched the functions of these hub genes (Fig. 8j). It was found that they were mainly related to the BP of “positive regulation of cytokine production”, “leukocyte migration”, “regulation of cell activation”, and “adaptive immune response”, as well as the molecular function (MF) of “CCR5 chemokine receptor activity and immune receptor activity”, and cellular component (CC) associations with “inner mitochondrial membrane protein complex”. The KEGG sulfur metabolism pathways underscore the critical processes involved in the “cytokine-cytokine receptor interaction” and the “toll-like receptor pathway” related to “primary immunodeficiency.” The proposed analytical framework, which integrates multi-dimensional data, elucidates the molecular mechanisms by which PtPb@SbO_3-x_ combats bacterial pneumonia through a dual-axis regulatory network of immune and inflammatory responses. This study offers novel insights for precise mechanism analysis in nanozyme therapy.

### 3.7 Biosafety assessment of PtPb@SbO_3-x_

The biosafety profile of PtPb@SbO_3-x_ was systematically evaluated through *in vitro* and *in vivo* investigations. The biocompatibility of PtPb@SbO_3-x_ was assessed in BEAS-2B human bronchial epithelial cells using the CCK-8 assay. The nanozyme exhibited no significant cytotoxicity at concentrations up to 200 ppm, with cell viability remaining above 75% regardless of the presence or absence of NIR-II irradiation (Fig. S30). These findings suggest that PtPb@SbO_3-x_ demonstrates excellent cytocompatibility, marking a crucial initial step in evaluating its *in vivo* biosafety. Hematological analyses, involving white blood cell (WBC), mean corpuscular hemoglobin (MCH), mean corpuscular volume (MCV), red blood cell (RBC), red blood cell distribution width (RDW), hemoglobin (HGB), mean corpuscular hemoglobin concentration (MCHC), platelet count (PLT), Monocyte (Mon), Granulocyte (Gran), hematocrit (HCT), were done for *in vitro* toxicity evaluation. The gamma-glutamyl transferase (GGT), creatine kinase (CK), blood urea nitrogen (BUN), aspartate aminotransferase (AST), and alanine aminotransferase (ALT) biochemical profiling studies were also performed (Fig. S31). Moreover, hemolysis assays were conducted to determine PtPb@SbO_3-x_ biocompatibility.

To systematically evaluate the long-term *in vivo* biosafety of the PtPb@SbO_3-x_, we monitored serum biochemical parameters, including GGT, CK, BUN, and ALT, in healthy mice 20 days following a single intraperitoneal injection (Fig. S32). These markers serve as crucial indicators of biliary excretory function, myocardial/skeletal muscle integrity, glomerular filtration capacity, and hepatocellular injury. The serum biochemical parameters in the PtPb@SbO_3-x_ treatment group exhibited no significant differences compared to the PBS control group (p > 0.05), with all measured values remaining within normal physiological ranges. These findings indicate that, even after an extended observation period of 20 days, no detectable damage to vital metabolic and excretory organs (i.e., liver and kidneys) or myocardial injury was associated with the nanozyme. This robust result complements our previous acute and subacute toxicity tests conducted over 48 h, demonstrating that PtPb@SbO_3-x_ possesses acceptable biocompatibility and overall systemic safety over prolonged periods, thereby enhancing its potential for future clinical translation. As evidenced in Fig. S33, an optimal concentration was maintained to produce no hemolysis up to 200 ppm, suggesting that PtPb@SbO_3-x_ possesses excellent biosafety. All parameters were within physiological range and did not differ significantly from baseline. The material has been shown to be hemocompatible and hepatorenal safe in experiments. Toxicological evaluation by histopathological analysis *in vivo* showed that the treated mice integrated tissue architecture in the vital organs (lung, liver, spleen, kidney, and heart) was preserved whereas inflammatory infiltrates, necrotic foci or any other pathological manifestations (Fig. S34). It is noteworthy that even when the nanomaterial was applied synergistically with the PTT and CDT modalities, no biosafety issues were detected. Body weight was monitored longitudinally throughout the experiment and after the therapeutic regimen, revealing consistent growth across all experimental groups. This further demonstrates the compatibility of material with the entire organism. The significant safety validation presented here establishes a robust preclinical foundation for advancing these nanoplatforms toward potential applications in combinatorial anti-infective therapies.

## 4 Conclusion

A defect/interface co-engineered PtPb@SbO_3-x_ nanozyme system has been developed through structural design. We systematically elucidate the unique structure and catalytic mechanisms of PtPb@SbO_3-x_ using a combination of advanced characterization techniques and theoretical simulations. Our findings demonstrate that the introduction of oxygen vacancy structures in PtPb@SbO_3-x_ results in a 1.6-fold enhancement in catalytic activity, accompanied by remarkable photothermal conversion efficiency (*η* = 58.7%) when irradiated with a 1064 nm laser. The metabolic profiler can effectively disrupt bacterial metabolism by regulating the ROS cascade, thereby inhibiting key enzymes in the TCA cycle pathway. In murine pneumonia models, the combined PTT and CDT achieved a remarkable 99.4% eradication rate of bacteria. This treatment was associated with a significant upregulation of the M2-polarization marker CD206, along with a marked downregulation of pro-inflammatory cytokines, including IL-6 and TNF-α in BALF. Collectively, these findings indicate a transition toward a reparative immune microenvironment and a suppression of pro-inflammatory markers such as CD86. A comprehensive analysis integrating multiple omics strategies, specifically transcriptomics and proteomics, elucidates the molecular cascade of inflammatory signaling pathways that coordinate both anti-infective and anti-inflammatory effects. This work establishes foundational principles for rational defect-engineered nanozyme design and proposes a multifunctional therapeutic strategy that interferes with metabolic and biochemical pathways to combat antibiotic-resistant infections. Despite these encouraging results, this study has several limitations. These include the reliance on a single murine pneumonia model, a relatively small sample size for each group, and the lack of long-term biodistribution and chronic toxicity evaluations. Future preclinical and translational studies should thoroughly investigate these aspects.

## CRediT authorship contribution statement

**Zhangwei Qiu:** Conceptualization, Writing – original draft. **Danyan Wang:** Conceptualization, Writing – original draft. **Zijun Jin:** Conceptualization, Data curation, Investigation. **Jiaping Han:** Formal analysis, Methodology. **Xueli Wang:** Methodology, Supervision. **Xiaojun He:** Funding acquisition, Project administration, Resources, Supervision. **Liqin Wu:** Funding acquisition, Writing – review & editing.

## Declaration of competing interest

The authors declare that they have no known competing financial interests or personal relationships that could have appeared to influence the work reported in this paper.

## Data Availability

Data will be made available on request.
