# Supplementary material for: Multi-omics decodes a defect-interface dual-engineered PtPb@SbO3-x nanozyme for NIR-II photothermal-amplified eradication of drug-resistant pneumonia
Source: Mater Today Bio. 2026 Aug 5;40:103535. doi: 10.1016/j.mtbio.2026.103535 (PMC13476549; doi:10.1016/j.mtbio.2026.103535)
Supplement: Supplementary file 1 [file mmc1.docx]

Supporting Information

Multi-omics decodes a defect-interface dual-engineered PtPb@SbO_3-x_ nanozyme for NIR-II photothermal-amplified eradication of drug-resistant pneumonia

Zhangwei Qiu,^a,#^ Danyan Wang,^e,#^ Zijun Jin,^b^ Jiaping Han,^c^ Xueli Wang,^c^ Xiaojun He,^d,*^ and Liqin Wu, ^a,*^

^a^ Department of Respiratory and Critical Care Medicine, The Second Affiliated Hospital and Yuying Children’s Hospital of Wenzhou Medical University, Wenzhou 325000, Zhejiang, China.

^b^ Zhejiang Chinese Medical University, Hangzhou, Zhejiang, 310000, China.

^c^ Department of Anesthesiology, The First Affiliated Hospital of Wenzhou Medical University, Wenzhou, Zhejiang, 325000, China.

^d^ Department of Ophthalmology, Peking University First Hospital, Beijing 100034, China.

^e^ Zhejiang Key Laboratory of Smart Biomaterials and Center for Bionanoengineering, College of Chemical and Biological Engineering, Zhejiang University, Hangzhou 310058, China.

All authors have approved the final version of this manuscript.

^#^These authors contributed equally to this study.

*Address correspondence: 21394@pkufh.com (X. He), [happy-qin@163.com](mailto:happy-qin@163.com) (L. Wu).

Figure S1. TEM-EDS spectra of PtPb@SbO_3-x_.

Figure S2. Raman spectra of PtPb@SbO_3-x_.

Figure S3. HAADF-STEM image with elemental mappings of PtPb@SbO_3-x_.

Figure S4. The corresponding fitting curve of the Fourier-transformed EXAFS spectrum of PtPb@SbO_3-x_.

Figure S5. EXAFS spectra of PtPb@SbO_3-x_.

Figure S6. (a) XPS survey spectra of PtPb@SbO_3-x_. High-resolution XPS for (b) Pb 4f, (c) C 1s, and (d) O 1s of PtPb@SbO_3-x_.

Figure S7. UV-vis-NIR absorption spectra of PtPb@SbO_3-x_ at different concentrations (0 to 200 ppm).

Figure S8. Infrared thermal images of water and PtPb@SbO_3-x_ (0-200 ppm) under irradiation (1064 nm, 1 W cm^-2^) at different times.

Figure S9. (a) The zeta potential and (b) particle size distribution of PtPb@SbO_3-x_ at 24 h in PBS (pH 7.4). (c) The particle size distribution and (d) zeta potential of PtPb@SbO_3-x_ at 48 h in PBS (pH 7.4). Each group was tested in triplicate.

Figure S10. Linear fitting plots of time versus ln (*θ*) during the cooling period.

Figure S11. Optimized structural configurations of TMB adsorbed on PtPb and PtPb@SbO_3-x_.

Figure S12. The work function of PtPb and PtPb@SbO_3-x_.

Figure S13. Calculated electrostatic potentials and the model structure of (a) PtPb and (b) PtPb@SbO_3-x_.

Figure S14. Photographs of the agar plates of the surviving MRSA at different concentrations (0 to 400 ppm).

Figure S15. The corresponding bacterial viability of MRSA with diﬀerent treatments.

Figure S16. Representative SEM images depict MRSA following various treatments.

Figure S17. The expression intensity heat map of 268 metabolites in MRSA.

Figure S18. Correlation heat map between samples.

Figure S19. Scatter plot of PCA principal component analysis.

Figure S20. Representative images of immunohistochemistry staining of (a) IL-10 and (b) VEGF on lung histology sections taken from different conditions.

Figure S21. Heat map of relative expression intensity of 13,991 identified mRNA in each sample.

Figure S22. Heat map of the relative expression intensity of 13,000 identified proteins in each sample.

Figure S23. The bar plot of transcriptomics GSEA BP TOP20 results.

Figure S24. The bar plot of proteomics GSEA BP TOP20 results.

Figure S25. Transcriptomics down-regulated the GO function enrichment BP TOP20 of the Hub gene.

Figure S26. Proteomics down-regulates the GO function enrichment BP TOP20 of the Hub gene.

Figure S27. Transcriptomically up-regulated PPI network map of DEGs and functional enrichment results of hub gene.

Figure S28. Proteomic up-regulation of DEGs PPI network map and TOP20 results of GO BP functional enrichment of hub gene.

Figure S29. Log_2_FC heat maps of two omics expressing opposite genes.

**Figure S30.** The in vitro cytotoxicity of PtPb@SbO_3-x_ was assessed by evaluating the viability of BEAS-2B cells with and without NIR-II irradiation (1.0 W cm^-2^, 6 min) after a 24-hour incubation period with PtPb@SbO_3-x_ at concentrations ranging from 0 to 200 ppm. The data presented are derived from three independent experiments (n = 3).

**Figure S31.** (a-k) Blood routine examination, including WBC, MCH, MCV, RBC, RDW, HGB, MCHC, PLT, Mon, Gran, and HCT. (l-p) Blood biochemistry examination, including GGT, CK, BUN, AST, and ALT of healthy mice received intraperitoneal injection of PBS or PtPb@SbO_3-x_ (100 ppm) after treatment with 48 h.

**Figure S32.** Blood biochemistry examination, including (a) GGT, (b) CK, (c) BUN, and (d) ALT of healthy mice received intraperitoneal injection of PBS or PtPb@SbO_3-x_ (100 ppm) after treatment with 20 days.

Figure S33. Representative photographs and quantitative analysis of hemolysis activity of PtPb@SbO_3-x_ dispersions with different concentrations.

**Figure S34.** H&E staining images of main organs (heart, liver, spleen, and kidney) of mice after receiving various therapies.

Table S1. Structural parameters of PtPb@SbP_3-x_ extracted from the EXAFS fitting. (So^2^=0.90)

| Sample | Shell | CN | R(Å) | Δσ^2^*10^3^ (Å^2^) | r-factor |
| --- | --- | --- | --- | --- | --- |
| PtPb@SbO_3-x_ | Pt-O | 4.8±1.6 | 2.03±0.01 | 8.76±1.31 | 0.022 |

S_o_^2^ is the amplitude reduction factor; CN is the coordination number; R is the interatomic distance (the bond length between central atoms and surrounding coordination atoms); σ^2^ is the Debye-Waller factor (a measure of thermal and static disorder in absorber-scatterer distances); R factor is used to value the goodness of the fitting.

Table S2. Photothermal conversion efficiencies of reported nanomaterials.

| Abbreviation | Description | PCE/η | Laser | Ref. |
| --- | --- | --- | --- | --- |
| Pt-NDs | Pt nanodots coated with BSA | 46.9% | 785 nm | [1] |
| cisPt@BRNPs | Black pigment gallstone-inspired platinum‐chelated bilirubin nanoparticles | 22.9 % | 808 nm | [2] |
| Pt-PEG | Worm-like Pt nanoparticles | 38.9% | 1064 nm | [3] |
| Pt NPs | five different-sized/shaped Pt nanoparticles | 22.98-30.88% | 1064 nm | [4] |
| Pt Cubes | Cubic shape solid Pt nanoparticles | 32.3% | 1120 nm | [5] |
| CuS NSs | copper-based nanoplatform | 44.1% | 1270 nm | [6] |
| Cu_3_BiS_3_ | Cu_3_BiS_3_ ternary semiconductor nanorods | 40.7% | 1064 nm | [7] |
| PtPb@SbO_3-x_ | PtPb and SbO_3-x_ heterojunction structure | 58.6% | 1064 nm | This work |

Table S3. Comparison of the kinetic parameters of PtPb@SbO_3-x_ and other nanozyme.

| Nanozyme | Substrates | K_m_ (10^-3^ M) | Vmax (10^-7^ M S^-1^) | Ref. |
| --- | --- | --- | --- | --- |
| Ir/NC SAC | H_2_O_2_ | 0.69 | 4.2 | [8] |
| CuS NSs | H_2_O_2_ | 1.36 | 3.1 | [6] |
| Mo_4_VC_4_ | H_2_O_2_ | 1.49 | 3.77 | [9] |
| Ag@V_2_C | H_2_O_2_ | 8.27 | 1.07 | [10] |
| PtPb@SbO_3-x_ | H_2_O_2_ | 0.47 | 3.0 | This work |

Table S4. The expression of 268 metabolites in MRSA.

| **Metabolites** | **Log_2_FC** | **P Value** | **adj.P.Val** |
| --- | --- | --- | --- |
| HMDB0000095 | 0.699363 | 4.81E-07 | 0.000121 |
| HMDB0000044 | 1.484563 | 9.00E-07 | 0.000121 |
| HMDB0000289 | 0.977919 | 1.84E-06 | 0.000137 |
| HMDB0000251 | -0.26016 | 2.43E-06 | 0.000137 |
| HMDB0000565 | -0.57285 | 2.55E-06 | 0.000137 |
| HMDB0000301 | 1.367368 | 6.15E-06 | 0.000275 |
| HMDB0000067 | 3.051218 | 2.18E-05 | 0.000835 |
| HMDB0002428 | -1.14819 | 3.43E-05 | 0.001149 |
| HMDB0000112 | -0.10635 | 4.29E-05 | 0.001255 |
| HMDB0000826 | -1.19778 | 4.68E-05 | 0.001255 |
| HMDB0000806 | -1.4579 | 8.49E-05 | 0.001937 |
| HMDB0000719 | -0.54952 | 8.67E-05 | 0.001937 |
| HMDB0000254 | -1.51485 | 0.000151 | 0.003113 |
| HMDB0000107 | 0.759126 | 0.000168 | 0.003224 |
| HMDB0000132 | -0.71318 | 0.000196 | 0.003509 |
| HMDB0003402 | -0.04314 | 0.000285 | 0.004775 |
| HMDB0000099 | 5.734447 | 0.000323 | 0.005099 |
| HMDB0000228 | 0.30204 | 0.000356 | 0.005305 |
| HMDB0000744 | 0.076718 | 0.000404 | 0.005697 |
| HMDB0000299 | -1.37143 | 0.000431 | 0.005769 |
| HMDB0000271 | -0.05657 | 0.000673 | 0.008583 |
| HMDB0062620 | -0.05106 | 0.000811 | 0.009051 |
| HMDB0001370 | -0.41576 | 0.000821 | 0.009051 |
| HMDB0001644 | -0.07095 | 0.000832 | 0.009051 |
| HMDB0003157 | 0.687159 | 0.000844 | 0.009051 |
| HMDB0000532 | -0.30461 | 0.001155 | 0.011908 |
| HMDB0001043 | -1.51672 | 0.00157 | 0.015586 |
| HMDB0000283 | -0.04368 | 0.001727 | 0.015647 |
| HMDB0000822 | 0.541832 | 0.001741 | 0.015647 |
| HMDB0010734 | -1.59282 | 0.001767 | 0.015647 |
| HMDB0061930 | -0.05098 | 0.00181 | 0.015647 |
| HMDB0000419 | 2.28567 | 0.001879 | 0.015735 |
| HMDB0000239 | 0.367245 | 0.002009 | 0.016313 |
| HMDB0061709 | 1.055418 | 0.002077 | 0.01637 |
| HMDB0000207 | 0.489926 | 0.002301 | 0.017617 |
| HMDB0002721 | -2.98439 | 0.002492 | 0.018554 |
| HMDB0000125 | 0.63234 | 0.003078 | 0.022296 |
| HMDB0000448 | 0.123616 | 0.003349 | 0.023616 |
| HMDB0000700 | -0.22596 | 0.003751 | 0.025774 |
| HMDB0003229 | -0.82754 | 0.004029 | 0.025829 |
| HMDB0000639 | 0.192973 | 0.004043 | 0.025829 |
| HMDB0000056 | -0.06689 | 0.004048 | 0.025829 |
| HMDB0000641 | -1.73079 | 0.004472 | 0.027874 |
| HMDB0000660 | 2.044604 | 0.005268 | 0.031834 |
| HMDB0014344 | -0.88906 | 0.005406 | 0.031834 |
| HMDB0000673 | -0.81932 | 0.005464 | 0.031834 |
| HMDB0000897 | -3.57413 | 0.005723 | 0.032488 |
| HMDB0000232 | 0.626699 | 0.005819 | 0.032488 |
| HMDB0000292 | 0.268308 | 0.006091 | 0.03323 |
| HMDB0001257 | -0.66232 | 0.0062 | 0.03323 |
| HMDB0000225 | -0.0523 | 0.006425 | 0.033765 |
| HMDB0000761 | 1.811138 | 0.007107 | 0.036626 |
| HMDB0000272 | 1.321082 | 0.007311 | 0.03697 |
| HMDB0000884 | 1.809358 | 0.00906 | 0.044965 |
| HMDB0011567 | 1.216626 | 0.009328 | 0.045454 |
| HMDB0003418 | -0.68933 | 0.010666 | 0.051006 |
| HMDB0000177 | -4.45667 | 0.011023 | 0.051006 |
| HMDB0000828 | -0.51736 | 0.011039 | 0.051006 |
| HMDB0015673 | 0.810121 | 0.012329 | 0.056003 |
| HMDB0001316 | 0.966808 | 0.01314 | 0.058693 |
| HMDB0001406 | -0.32585 | 0.0134 | 0.05887 |
| HMDB0029915 | 0.706189 | 0.014951 | 0.064378 |
| HMDB0013713 | -0.83513 | 0.015134 | 0.064378 |
| HMDB0004610 | -2.25783 | 0.016532 | 0.069227 |
| HMDB0002545 | 0.651289 | 0.017533 | 0.07229 |
| HMDB0061707 | 0.470401 | 0.018896 | 0.075333 |
| HMDB0000929 | -0.4219 | 0.019101 | 0.075333 |
| HMDB0000618 | 1.00351 | 0.019114 | 0.075333 |
| HMDB0000288 | -0.65445 | 0.021102 | 0.080554 |
| HMDB0003338 | -0.36405 | 0.021347 | 0.080554 |
| HMDB0003411 | -1.55323 | 0.021571 | 0.080554 |
| HMDB0000008 | -0.20165 | 0.022191 | 0.080554 |
| HMDB0004215 | 0.936034 | 0.022205 | 0.080554 |
| HMDB0001855 | -3.50881 | 0.022242 | 0.080554 |
| HMDB0002643 | -0.36537 | 0.024789 | 0.088579 |
| HMDB0000048 | -1.17906 | 0.027701 | 0.097455 |
| HMDB0003070 | -0.73028 | 0.028 | 0.097455 |
| HMDB0001451 | 0.543879 | 0.028723 | 0.098689 |
| HMDB0001209 | 0.162545 | 0.03064 | 0.103544 |
| HMDB0000115 | -0.20272 | 0.030909 | 0.103544 |
| HMDB0000168 | -0.50485 | 0.035004 | 0.115816 |
| HMDB0001147 | 0.174424 | 0.035524 | 0.116104 |
| HMDB0000827 | 1.570738 | 0.037064 | 0.119676 |
| HMDB0002039 | 0.070561 | 0.038468 | 0.122731 |
| HMDB0000172 | -1.26717 | 0.041965 | 0.132313 |
| HMDB0000355 | -0.06909 | 0.044244 | 0.137878 |
| HMDB0000143 | 0.438378 | 0.047952 | 0.146845 |
| HMDB0000190 | -0.52192 | 0.048218 | 0.146845 |
| HMDB0031321 | -0.60609 | 0.049996 | 0.150551 |
| HMDB0000296 | 1.575825 | 0.051367 | 0.15296 |
| HMDB0002712 | -0.28622 | 0.054583 | 0.159559 |
| HMDB0003747 | 1.118621 | 0.054774 | 0.159559 |
| HMDB0002068 | 1.220101 | 0.057218 | 0.164885 |
| HMDB0000423 | 0.845032 | 0.059271 | 0.168987 |
| HMDB0001238 | 0.541847 | 0.060178 | 0.169766 |
| HMDB0000159 | 0.225744 | 0.061394 | 0.170165 |
| HMDB0000671 | -0.49936 | 0.06159 | 0.170165 |
| HMDB0029943 | -0.50947 | 0.06648 | 0.181803 |
| HMDB0000045 | -0.6532 | 0.068843 | 0.185759 |
| HMDB0039721 | 1.443811 | 0.069313 | 0.185759 |
| HMDB0000696 | 1.156081 | 0.070567 | 0.186736 |
| HMDB0001514 | -0.44993 | 0.071071 | 0.186736 |
| HMDB0094692 | -0.36957 | 0.072799 | 0.188906 |
| HMDB0001227 | 0.350257 | 0.07393 | 0.188906 |
| HMDB0000089 | -0.54578 | 0.074012 | 0.188906 |
| HMDB0000161 | -0.4294 | 0.076998 | 0.193211 |
| HMDB0000484 | -0.32041 | 0.07714 | 0.193211 |
| HMDB0000803 | 0.533 | 0.082614 | 0.205005 |
| HMDB0011747 | -1.5532 | 0.083812 | 0.206069 |
| HMDB0001047 | -0.33123 | 0.086669 | 0.211158 |
| HMDB0001129 | -1.86061 | 0.092171 | 0.221367 |
| HMDB0000210 | -0.44154 | 0.093197 | 0.221367 |
| HMDB0003119 | -0.17066 | 0.093338 | 0.221367 |
| HMDB0011533 | 0.653979 | 0.094324 | 0.221745 |
| HMDB0001388 | -0.57653 | 0.096407 | 0.224671 |
| HMDB0000202 | -0.02303 | 0.116109 | 0.268252 |
| HMDB0003072 | -0.29418 | 0.118988 | 0.271981 |
| HMDB0002345 | 0.356999 | 0.119753 | 0.271981 |
| HMDB0000163 | 0.537094 | 0.127429 | 0.286982 |
| HMDB0000562 | -0.03891 | 0.132483 | 0.29426 |
| HMDB0000072 | -0.52341 | 0.132856 | 0.29426 |
| HMDB0003164 | 1.196277 | 0.139466 | 0.306368 |
| HMDB0002064 | -0.33666 | 0.145203 | 0.315061 |
| HMDB0000247 | -0.95912 | 0.145774 | 0.315061 |
| HMDB0001448 | 0.118173 | 0.151338 | 0.324469 |
| HMDB0000124 | 0.043656 | 0.154026 | 0.32761 |
| HMDB0000290 | 0.838482 | 0.156991 | 0.331288 |
| HMDB0003540 | 0.705381 | 0.166596 | 0.34881 |
| HMDB0011131 | 0.428839 | 0.168823 | 0.350733 |
| HMDB0004041 | -0.0108 | 0.175236 | 0.361257 |
| HMDB0000516 | -0.35256 | 0.177298 | 0.362717 |
| HMDB0000687 | 0.829627 | 0.179384 | 0.364204 |
| HMDB0000157 | 0.237027 | 0.183388 | 0.369534 |
| HMDB0000094 | -0.50804 | 0.18614 | 0.372281 |
| HMDB0000511 | -0.20683 | 0.192971 | 0.383082 |
| HMDB0001492 | -0.97184 | 0.195469 | 0.385189 |
| HMDB0000262 | 0.116825 | 0.199159 | 0.388136 |
| HMDB0000772 | -0.62163 | 0.199861 | 0.388136 |
| HMDB0001964 | 0.146634 | 0.203503 | 0.392366 |
| HMDB0000881 | -2.44526 | 0.206383 | 0.395076 |
| HMDB0000904 | 1.144939 | 0.212365 | 0.403643 |
| HMDB0000214 | -0.28687 | 0.214356 | 0.40374 |
| HMDB0013682 | -1.67825 | 0.215428 | 0.40374 |
| HMDB0000840 | -1.0744 | 0.21858 | 0.405515 |
| HMDB0000573 | 0.719909 | 0.219402 | 0.405515 |
| HMDB0000252 | 0.469491 | 0.222508 | 0.408439 |
| HMDB0000764 | -0.08141 | 0.225897 | 0.411839 |
| HMDB0028853 | -0.58852 | 0.227445 | 0.411861 |
| HMDB0000167 | 0.017023 | 0.23095 | 0.4154 |
| HMDB0000164 | -0.20448 | 0.246793 | 0.440936 |
| HMDB0001548 | 0.85954 | 0.251498 | 0.445352 |
| HMDB0000195 | 1.107416 | 0.252819 | 0.445352 |
| HMDB0012275 | -0.0179 | 0.254249 | 0.445352 |
| HMDB0002362 | 0.090665 | 0.26636 | 0.463536 |
| HMDB0000267 | 0.219222 | 0.269298 | 0.465625 |
| HMDB0000076 | 0.009316 | 0.276609 | 0.475201 |
| HMDB0002994 | 0.045698 | 0.283558 | 0.482178 |
| HMDB0001857 | 0.204039 | 0.285274 | 0.482178 |
| HMDB0000182 | 1.044741 | 0.286069 | 0.482178 |
| HMDB0040937 | 0.77984 | 0.295911 | 0.493293 |
| HMDB0000131 | -0.10546 | 0.296344 | 0.493293 |
| HMDB0000704 | -0.22514 | 0.300454 | 0.496054 |
| HMDB0000019 | 0.011119 | 0.301987 | 0.496054 |
| HMDB0001313 | 0.729571 | 0.303556 | 0.496054 |
| HMDB0000279 | 0.318084 | 0.312539 | 0.507639 |
| HMDB0003355 | -0.08001 | 0.316262 | 0.510592 |
| HMDB0003447 | -0.31526 | 0.328771 | 0.527608 |
| HMDB0000801 | -0.74508 | 0.332789 | 0.530877 |
| HMDB0000306 | -1.31538 | 0.347489 | 0.551047 |
| HMDB0001076 | -0.25392 | 0.353567 | 0.557389 |
| HMDB0000150 | -0.32719 | 0.362698 | 0.566655 |
| HMDB0000226 | 0.586626 | 0.364765 | 0.566655 |
| HMDB0000638 | 0.006658 | 0.365789 | 0.566655 |
| HMDB0002432 | 0.022747 | 0.369912 | 0.569291 |
| HMDB0002329 | -0.06555 | 0.371738 | 0.569291 |
| HMDB0002085 | -0.68418 | 0.380246 | 0.579011 |
| HMDB0000721 | 0.243842 | 0.382584 | 0.57928 |
| HMDB0001256 | 0.380659 | 0.406712 | 0.612353 |
| HMDB0000158 | -0.85298 | 0.409531 | 0.613153 |
| HMDB0000133 | 0.333325 | 0.412469 | 0.61412 |
| HMDB0000175 | 0.369842 | 0.418646 | 0.619874 |
| HMDB0030637 | 0.026785 | 0.433095 | 0.637744 |
| HMDB0001488 | 0.008393 | 0.438302 | 0.641885 |
| HMDB0002923 | 0.275182 | 0.441603 | 0.643204 |
| HMDB0000169 | 0.338413 | 0.45612 | 0.660758 |
| HMDB0000765 | -0.49187 | 0.46031 | 0.663243 |
| HMDB0000714 | -0.20349 | 0.463856 | 0.664777 |
| HMDB0000883 | -0.13106 | 0.477596 | 0.680828 |
| HMDB0000259 | 0.213677 | 0.481737 | 0.683018 |
| HMDB0002928 | 0.194328 | 0.486422 | 0.683018 |
| HMDB0000613 | 0.006896 | 0.486778 | 0.683018 |
| HMDB0000867 | 0.132058 | 0.495147 | 0.691143 |
| HMDB0002356 | 0.719677 | 0.500835 | 0.69546 |
| HMDB0000193 | -0.81408 | 0.509587 | 0.703966 |
| HMDB0000767 | -0.37408 | 0.513304 | 0.705464 |
| HMDB0000216 | -0.32921 | 0.531391 | 0.726596 |
| HMDB0000192 | 0.370391 | 0.539537 | 0.73399 |
| HMDB0000243 | -0.07141 | 0.556118 | 0.752725 |
| HMDB0001856 | 0.131827 | 0.563766 | 0.759242 |
| HMDB0033871 | 0.007791 | 0.573232 | 0.762996 |
| HMDB0000186 | -0.15939 | 0.57501 | 0.762996 |
| HMDB0000710 | -0.0121 | 0.575094 | 0.762996 |
| HMDB0000609 | 0.115371 | 0.584239 | 0.767705 |
| HMDB0011537 | 0.338329 | 0.58627 | 0.767705 |
| HMDB0011749 | -0.01124 | 0.588442 | 0.767705 |
| HMDB0001124 | -0.3435 | 0.590102 | 0.767705 |
| HMDB0062238 | -0.2425 | 0.603754 | 0.781671 |
| HMDB0000740 | -0.14818 | 0.614459 | 0.791706 |
| HMDB0001414 | 0.238352 | 0.627645 | 0.804828 |
| HMDB0000975 | 0.148397 | 0.634268 | 0.809447 |
| HMDB0029196 | -0.00362 | 0.640724 | 0.811491 |
| HMDB0002649 | -0.02356 | 0.641926 | 0.811491 |
| HMDB0000017 | -0.40262 | 0.66382 | 0.834888 |
| HMDB0000625 | 0.205878 | 0.666664 | 0.834888 |
| HMDB0002117 | 0.19671 | 0.680599 | 0.848375 |
| HMDB0000258 | 0.220469 | 0.699987 | 0.860567 |
| HMDB0000197 | -0.29365 | 0.700152 | 0.860567 |
| HMDB0000126 | -0.2034 | 0.703613 | 0.860567 |
| HMDB0001138 | 0.085849 | 0.703782 | 0.860567 |
| HMDB0001923 | -0.06732 | 0.706436 | 0.860567 |
| HMDB0003357 | 0.089558 | 0.712566 | 0.864107 |
| HMDB0000645 | -0.23636 | 0.723846 | 0.870165 |
| HMDB0001870 | -0.00517 | 0.724055 | 0.870165 |
| HMDB0001401 | 0.021119 | 0.734114 | 0.878315 |
| HMDB0000715 | -0.07145 | 0.738586 | 0.879685 |
| HMDB0002350 | 0.131568 | 0.741824 | 0.879685 |
| HMDB0000211 | 0.039125 | 0.750941 | 0.883572 |
| HMDB0000224 | 0.050927 | 0.751695 | 0.883572 |
| HMDB0000944 | 0.133257 | 0.773415 | 0.900151 |
| HMDB0141542 | -0.15887 | 0.784205 | 0.900151 |
| HMDB0062802 | 0.004506 | 0.785825 | 0.900151 |
| HMDB0005826 | 0.226978 | 0.787282 | 0.900151 |
| HMDB0014724 | -0.04722 | 0.787426 | 0.900151 |
| HMDB0033944 | 0.005076 | 0.791149 | 0.900151 |
| HMDB0001078 | -0.12231 | 0.792556 | 0.900151 |
| HMDB0003911 | 0.017392 | 0.795983 | 0.900151 |
| HMDB0011564 | -0.13201 | 0.798135 | 0.900151 |
| HMDB0002199 | -0.04745 | 0.802148 | 0.900151 |
| HMDB0001392 | -0.03534 | 0.802747 | 0.900151 |
| HMDB0000118 | 0.043993 | 0.816615 | 0.911886 |
| HMDB0000807 | -0.30196 | 0.820589 | 0.912522 |
| HMDB0000220 | 0.021666 | 0.834911 | 0.921353 |
| HMDB0001264 | 0.035221 | 0.835406 | 0.921353 |
| HMDB0001893 | 0.123373 | 0.841257 | 0.924003 |
| HMDB0000634 | -0.00484 | 0.84674 | 0.926229 |
| HMDB0034219 | -0.03227 | 0.859305 | 0.936153 |
| HMDB0001058 | 0.140799 | 0.873287 | 0.945324 |
| HMDB0038361 | -0.06411 | 0.87808 | 0.945324 |
| HMDB0014457 | -0.03329 | 0.879394 | 0.945324 |
| HMDB0003156 | -0.02913 | 0.881832 | 0.945324 |
| HMDB0004095 | 0.037399 | 0.902566 | 0.962326 |
| HMDB0000847 | 0.021266 | 0.910958 | 0.962326 |
| HMDB0000212 | 0.021266 | 0.910958 | 0.962326 |
| HMDB0000055 | -0.02736 | 0.912432 | 0.962326 |
| HMDB0001999 | -0.04606 | 0.918168 | 0.962326 |
| HMDB0000575 | 0.032563 | 0.922405 | 0.962326 |
| HMDB0002019 | 0.042832 | 0.925677 | 0.962326 |
| HMDB0000684 | 0.024498 | 0.926419 | 0.962326 |
| HMDB0000034 | 0.006409 | 0.93209 | 0.962692 |
| HMDB0001624 | -0.00279 | 0.933955 | 0.962692 |
| HMDB0000725 | -0.00146 | 0.955231 | 0.978874 |
| HMDB0000149 | -0.00143 | 0.960159 | 0.978874 |
| HMDB0028832 | 0.011694 | 0.960612 | 0.978874 |
| HMDB0000663 | -0.04047 | 0.966996 | 0.981647 |
| HMDB0003331 | -0.00836 | 0.980071 | 0.991166 |
| HMDB0000085 | 0.019499 | 0.988318 | 0.995749 |
| HMDB0000050 | -0.00257 | 0.99362 | 0.996653 |
| HMDB0002338 | -0.00137 | 0.996653 | 0.996653 |

Table S5. Relative expression of 13,991 identified mRNA in each sample.

|  | LogFC | LogCPM | P_Value_ | FDR |
| --- | --- | --- | --- | --- |
| Dmbt1 | -5.70214 | 2.856976 | 7.27E-14 | 1.02E-09 |
| Fabp4 | -3.06684 | 7.016505 | 2.79E-13 | 1.95E-09 |
| Cd8a | 2.231732 | 1.697967 | 5.00E-10 | 2.33E-06 |
| Ifi44 | -1.76677 | 3.532574 | 8.58E-10 | 3.00E-06 |
| Cidec | -1.79931 | 4.474268 | 2.31E-09 | 6.46E-06 |
| A2ml1 | -2.44225 | 4.812104 | 4.18E-09 | 9.74E-06 |
| Ltf | -1.89894 | 7.165 | 2.79E-08 | 5.58E-05 |
| Tmem45b | -4.44476 | 0.961262 | 6.43E-08 | 0.000103 |
| Ifitm1 | -1.29743 | 7.072641 | 7.19E-08 | 0.000103 |
| Cd3d | 1.755623 | 2.085618 | 7.37E-08 | 0.000103 |
| Lep | -3.7947 | 0.009103 | 8.72E-08 | 0.000111 |
| Ifi202b | -1.43337 | 3.641211 | 1.22E-07 | 0.000142 |
| Ms4a6d | -1.84377 | 5.025885 | 1.65E-07 | 0.000177 |
| Col10a1 | -8.51241 | 1.285416 | 1.88E-07 | 0.000188 |
| Plin1 | -3.29554 | 2.874398 | 3.01E-07 | 0.000281 |
| Serpinb11 | -4.35097 | 0.166214 | 4.17E-07 | 0.000365 |
| Cxcl2 | -5.67057 | 5.310792 | 5.10E-07 | 0.00042 |
| Cxcl13 | -2.65641 | 4.726512 | 6.35E-07 | 0.000491 |
| Acta1 | -3.38839 | 0.915477 | 6.66E-07 | 0.000491 |
| Pck1 | -4.18318 | 4.226656 | 1.33E-06 | 0.000932 |
| Ddit4 | -1.11633 | 5.536032 | 1.45E-06 | 0.000965 |
| Cd177 | -1.76339 | 6.439465 | 1.61E-06 | 0.001022 |
| Pglyrp1 | -1.02209 | 5.843114 | 1.78E-06 | 0.001055 |
| Wnt9b | -2.05663 | 1.095847 | 1.81E-06 | 0.001055 |
| Cr2 | 2.476746 | 1.444322 | 1.92E-06 | 0.001075 |
| Vcan | -2.33473 | 4.55838 | 2.00E-06 | 0.001076 |
| Bpifa1 | -2.05615 | 9.3612 | 2.66E-06 | 0.001333 |
| Msr1 | -2.52187 | 4.148445 | 2.67E-06 | 0.001333 |
| Cd79b | 0.992215 | 3.838858 | 2.89E-06 | 0.001392 |
| Tgfbi | -1.14761 | 7.180192 | 3.24E-06 | 0.0015 |

Table S6. Relative expression of 13,000 identified proteins in each sample.

|  | LogFC | LogCPM | P_Value_ | FDR |
| --- | --- | --- | --- | --- |
| Saa3 | -10.8847 | 9.90253 | 2.06E-106 | 2.68E-102 |
| Ccl8 | -6.2288 | 6.613205 | 1.59E-60 | 1.03E-56 |
| Clec4d | -5.98279 | 8.237787 | 5.98E-52 | 2.59E-48 |
| Calhm6 | -4.66872 | 5.786151 | 3.47E-47 | 1.13E-43 |
| Cxcl16 | -5.27082 | 8.306527 | 3.37E-43 | 8.77E-40 |
| Slpi | -5.51378 | 10.16278 | 5.89E-43 | 1.28E-39 |
| Ccl20 | -11.3092 | 3.720912 | 4.45E-42 | 8.27E-39 |
| H2-Aa | -4.3392 | 9.149342 | 5.21E-42 | 8.47E-39 |
| Ctss | -4.40036 | 9.341774 | 7.91E-42 | 1.14E-38 |
| Clec4n | -6.10132 | 7.958432 | 2.51E-38 | 3.26E-35 |
| Slc7a11 | -6.58881 | 6.447558 | 4.29E-37 | 5.08E-34 |
| Ms4a6d | -4.58612 | 8.167103 | 2.79E-36 | 3.02E-33 |
| C1s1 | -3.69316 | 6.738486 | 1.16E-34 | 1.16E-31 |
| Ly86 | -2.99057 | 6.352032 | 3.32E-33 | 2.94E-30 |
| Clec4e | -6.28519 | 8.387318 | 3.39E-33 | 2.94E-30 |
| Dram1 | -3.21485 | 7.30534 | 6.53E-33 | 5.30E-30 |
| Fcgr1 | -3.17349 | 5.969553 | 8.90E-33 | 6.81E-30 |
| Cxcl13 | -10.3365 | 6.908675 | 1.18E-32 | 8.50E-30 |
| Cd300c2 | -3.58935 | 6.436505 | 3.82E-32 | 2.62E-29 |
| Satb2 | 3.385653 | 7.351861 | 6.67E-32 | 4.34E-29 |
| Arg1 | -7.7984 | 6.333597 | 2.80E-31 | 1.73E-28 |
| Cxcl9 | -6.72638 | 9.121702 | 6.82E-31 | 4.03E-28 |
| H2-DMb2 | -4.00101 | 6.673756 | 9.57E-31 | 5.41E-28 |
| Cd74 | -3.72539 | 9.99601 | 1.12E-30 | 6.09E-28 |
| Aif1 | -3.78951 | 6.890598 | 2.14E-30 | 1.11E-27 |
| Ltb | -3.52116 | 8.315139 | 2.77E-30 | 1.38E-27 |
| S100a6 | -3.25262 | 7.991655 | 3.43E-30 | 1.65E-27 |
| Adgrd1 | 3.304547 | 5.599238 | 6.68E-30 | 3.10E-27 |
| Il6 | -5.70541 | 4.530461 | 1.08E-29 | 4.83E-27 |
| Clec4a3 | -3.01494 | 6.057295 | 2.37E-28 | 1.03E-25 |

**References**

[1] Y. Tang, T. Yang, Q. Wang, X. Lv, X. Song, H. Ke, Z. Guo, X. Huang, J. Hu, Z. Li, Albumin-coordinated assembly of clearable platinum nanodots for photo-induced cancer theranostics, Biomaterials 154 (2018) 248-260.

[2] D.Y. Lee, J.Y. Kim, Y. Lee, S. Lee, W. Miao, H.S. Kim, J.J. Min, S. Jon, Black pigment gallstone inspired platinum‐chelated bilirubin nanoparticles for combined photoacoustic imaging and photothermal therapy of cancers, Angew. Chem. Int. Ed. 129 (2017) 13872-13876.

[3] Q. Ma, L. Cheng, F. Gong, Z. Dong, C. Liang, M. Wang, L. Feng, Y. Li, Z. Liu, C. Li, Platinum nanoworms for imaging-guided combined cancer therapy in the second near-infrared window, J. Mater.Chem. B 6 (2018) 5069-5079.

[4] M. Manikandan, N. Hasan, H.-F. Wu, Platinum nanoparticles for the photothermal treatment of Neuro 2A cancer cells, Biomaterials 34 (2013) 5833-5842.

[5] Q. Wang, H. Wang, Y. Yang, L. Jin, Y. Liu, Y. Wang, X. Yan, J. Xu, R. Gao, P. Lei, Plasmonic Pt superstructures with boosted near‐infrared absorption and photothermal conversion efficiency in the second biowindow for cancer therapy, Adv. Mater. 31 (2019) 1904836.

[6] D. Wang, X. He, Q. Wang, P. Li, E. Obeng, D. Zheng, J. Shen, Z. Jin, NIR-II photo-amplified peroxidase-like activity using biocatalytic copper-based nanoplatform against subcutaneous methicillin-resistant *Staphylococcus aureus* infection, Chem. Eng. J. 471 (2023) 144449.

[7] A. Li, X. Li, X. Yu, W. Li, R. Zhao, X. An, D. Cui, X. Chen, W. Li, Synergistic thermoradiotherapy based on PEGylated Cu_3_BiS_3_ ternary semiconductor nanorods with strong absorption in the second near-infrared window, Biomaterials 112 (2017) 164-175.

[8] D. Wang, H. Jin, Y. Shen, D. Wang, J. He, J. Qu, X. He, Z. Jiang, NIR-II-Activated iridium single-atom nanozymes for synergistic antibacterial therapy and tissue regeneration in MRSA-infected wounds and acute lung injury, Bioact. Mater. 51 (2025) 543-558.

[9] Y. Liu, X. He, J. Feng, D. Wang, E. Obeng, C. Yu, Y. Song, J. Shen, Z. Li, Engineering a new member of MXenes M_5_C_4_ phases nanoplatforms as synergistically photothermal and chemodynamic therapeutics for methicillin-resistant *Staphylococcus aureus*, Chem. Eng. J. 466 (2023) 143004.

[10] X. Qian, H. Hou, Y. Wei, L. Zhang, Y. Zhai, G. Jiang, L. Liu, Z. Xiang, X. Tao, X. Liao, X. He, Metal-regulated MXene nanozyme with second near-infrared-triggered photothermal and chemodynamic synergy for anti-infection therapy and transcriptomic deciphering of antibacterial mechanisms, J. Colloid Interface Sci. 724 (2026) 141032.
